# Molecular action of isoflavone genistein in the human epithelial cell line HaCaT

**DOI:** 10.1371/journal.pone.0192297

**Published:** 2018-02-14

**Authors:** Elwira Smolińska, Marta Moskot, Joanna Jakóbkiewicz-Banecka, Grzegorz Węgrzyn, Bogdan Banecki, Aneta Szczerkowska-Dobosz, Dorota Purzycka-Bohdan, Magdalena Gabig-Cimińska

**Affiliations:** 1 Department of Medical Biology and Genetics, University of Gdańsk, Gdańsk, Poland; 2 Department of Physiology, Medical University of Gdańsk, Gdańsk, Poland; 3 Institute of Biochemistry and Biophysics, Polish Academy of Sciences, Laboratory of Molecular Biology, Gdańsk, Poland; 4 Department of Molecular Biology, University of Gdańsk, Gdańsk, Poland; 5 Department of Molecular and Cellular Biology, Intercollegiate Faculty of Biotechnology UG-MUG, Gdańsk, Poland; 6 Department of Dermatology, Venereology and Allergology, Medical University of Gdańsk, Gdańsk, Poland; University of Catanzaro, ITALY

## Abstract

Due to its strong proliferation-reducing effects on keratinocytes, and also anti-inflammatory properties, the isoflavone genistein has already been proposed as a possible antipsoriatic compound. As there is still no detailed information on this topic, we examined the effects of genistein by using an *in vitro* model of both, normal and “psoriasis-like” keratinocytes at this stage of our work exhaustively testing the selected flavonoid in a mono-treated experimental design. Gene expression studies revealed transcriptional changes that confirms known disease-associated pathways and highlights many psoriasis-related genes. Our results suggested that aberrant expression of genes contributing to the progress of psoriasis could be improved by the action of genistein. Genistein prevented “cytokine mix” as well as TNF-α-induced NF-κB nuclear translocation, with no effect on the PI3K signaling cascade, indicating the luck of turning this pathway into NF-κB activation. It could have attenuated TNF-α and LPS-induced inflammatory responses by suppressing ROS activation. Regardless of the type of keratinocyte stimulation used, reduction of cytokine IL-8, IL-20 and CCL2 production (both at RNA and protein level) following genistein treatment was visible. Because investigations of other groups supported our commentary on potential administration of genistein as a potential weapon in the armamentarium against psoriasis, it is believed that this paper should serve to encourage researchers to conduct further studies on this subject.

## Introduction

A broad spectrum of natural compounds is routinely applied in the daily diet of many patients with no information about possible mechanisms of their actions. Such agents with anti-inflammatory activities are applied all over the world as alternative medicines for psoriasis (Ps) because of their perceived beneficial impact on the skin. Although many treatments are available to reduce the bothersome symptoms and appearance of psoriasis, it continues to remain incurable, and its cause stays unelucidated. Within the last decade, substantial advances have been made in clarifying the molecular pathogenesis of psoriasis. Transcriptomic analyses have been widely used in recent years to identify differentially expressed genes (DEGs) associated with psoriasis pathology [1–6]. While there is now increasing insight into the genes conferring disease susceptibility, much less is known about the types of regulatory networks of expressed genes that define the molecular signature of the disease. Besides important mRNA expression alterations, Ps is also characterized by a specific microRNA expression profile, distinct from that of healthy skin [7]. In addition, the meta-analysis produced a reference list of consistent candidate genes for further investigation of psoriasis pathology and new therapeutic target selection with the use of genetic markers [4].

Genistein (4,5,7-trihydroxyisoflavone) is a naturally occurring plant compound that exhibits multidirectional biological action. It displays antioxidant, antiproliferative, proapoptotic, antiangiogenic, as well as estrogenic and anti-estrogenic activity. Ito’s group [8] investigated topical application of Glyteer (GL, soybean) on a psoriatic model in mice. This isoflavone has also attracted attention as a potent agent in the treatment of Ps [9, 10] not only due to its anti-proliferative and immunosuppressive properties but also as a mediator modulating expression of various genes whose products are involved among others in different phases of inflammation and proliferation [11]. However, the explicit mechanism of genistein’s action, especially in human epithelial cells, is still not elucidated. Thus, we examined the effects of genistein on activated spontaneously immortalized human keratinocytes, “psoriasis-like” HaCaT cell line, to find new potential targets for therapy and/or to develop a tool for treatment. Despite being limited by the lack of many of the cellular players in psoriasis, including fibroblasts and inflammatory cells, our monolayer *in vitro* model of “psoriasis-like” HaCaT has proved to be a valuable first step model system in the evaluation of nutraceutical agents such as genistein for Ps improvement [12–16]. Our results suggest that the aberrant expression of genes contributing to the progression of Ps can be improved by the action of genistein; they also explain in detail the effects of this isoflavone on signaling cascades in the human epithelial cell line HaCaT.

## Materials and methods

### Cell lines, culture media and reagents

Spontaneously immortalized human keratinocytes (HaCaT cell line) were purchased from the CLS Cell Lines Service GmbH (Eppelheim, Germany) [17]. HaCaT cells from passages 35–50 were maintained in Roswell Park Memorial Institute 1640 medium (RPMI 1640, Gibco, Thermo Fisher Scientific, Bleiswijk, Netherlands) supplemented with 10% fetal bovine serum (FBS, Gibco, Thermo Fisher Scientific, Bleiswijk, Netherlands) and 1% antibiotic/antimycotic solution (Gibco, Thermo Fisher Scientific, Bleiswijk, Netherlands) at 37°C in a humidified atmosphere containing 5% carbon dioxide (CO_2_). THP-1 cells (CLS Cell Lines Service GmbH) were routinely maintained in RPMI 1640 medium supplemented with 2 mM glutamine (Gibco, Thermo Fisher Scientific, Bleiswijk, Netherlands) and 10% FBS (Gibco, Thermo Fisher Scientific, Bleiswijk, Netherlands) at 2–8 x 10^5^ cells/mL. Genistein was synthetized at the Pharmaceutical Research Institute (Warsaw, Poland). The flavonoid was dissolved in dimethyl sulfoxide (DMSO, Sigma-Aldrich, St. Louis, USA) and added in the indicated final concentrations as determined in previous studies [18, 19] to cell cultures. Methotrexate (MTX) was purchased from Sigma-Aldrich (St. Louis, USA). It was dissolved in DMSO and added in the indicated final concentrations [20].

### Cell-viability assays

Cell viability was evaluated by MTT (3-(4,5-dimethylthiazol-2-yl)-2,5-diphenyltetrazolium bromide) assay. For cell cytotoxic assay and proliferation assay, 1.25 x 10^4^ or 10^3^ HaCaT cells, respectively, were seeded per well in 96-well culture plates and incubated overnight. Growth medium was then substituted with fresh medium supplemented with tested compounds at appropriate concentrations or 0.05% DMSO as a control (in cultures with tested compounds, DMSO final concentration was also adjusted to 0.05%). Following incubation for 24 and 48 hours (to test cytotoxicity) or for 7 -days (to test cell proliferation), medium was substituted with MTT solution (1 mg/mL in RPMI 1640 medium, Sigma-Aldrich, St. Louis, USA); and after a 2-hour incubation at 37°C the formazan product was dissolved in DMSO and absorbance was read at 550 nm (VICTOR Multilabel Plate Reader, PerkinElmer). The optical density of formazan formed in the control was taken as 100% of cell viability. Samples were measured in quintuplicate, and the experiment was repeated three times. LC (cytotoxicity assay) and IC (proliferation assay) index values of genistein was determined for each compound in comparison to non-treated cultures (incubated with DMSO only).

### Two-dimensional (2D) engineered skin psoriatic cells model development

#### Direct psoriatic stimulation

HaCaT cells were cultured to 80% of confluence in flasks containing RPMI medium supplemented with 10% FBS and 1% antibiotic/antimycotic solution and then the cells were disaggregated using 0.025% trypsin with 0.01% EDTA. Cells were seeded at a density of 4 x 10^5^ cells/well in 6-well plates in defined serum-free defined keratinocyte medium (Keratinocyte-SFM, Gibco, Thermo Fisher Scientific, CA, USA) supplemented with a pituitary extract including insulin (BPE) and epidermal growth factor (EGF) (Gibco, Thermo Fisher Scientific, CA, USA). After a 24-hour incubation cells were cultivated in serum-free keratinocyte medium without growth supplements for 16 hours. For further experiments, cells were stimulated with a combination of a proinflammatory “cytokine mix”: IL-1A, IL-17A, IL-22, oncostatin M (OSM), and tumor necrosis factor-α (TNF-α) (Gibco, Thermo Fisher Scientific, CA, USA) 2 ng/mL each or lipopolysaccharide (LPS, from *Escherichia coli 055*:*B5*, Sigma-Aldrich, St. Louis, USA) 1 μg/mL in the presence or absence of genistein for 24 hours [21]. The control cells were left untreated.

#### Indirect psoriatic stimulation

The Transwell cell culture system was used to co-culture THP-1 with HaCaT. THP-1 cells (3 x 10^6^) were cultured in the upper compartment in cell culture membrane inserts (Corning #353102), and HaCaT cells (4 x 10^5^) were cultured in the lower compartment in 6-well plates, both cell lines in RPMI medium supplemented with 10% FBS and 1% antibiotic/antimycotic solution. For the co-cultivation studies, HaCaT cells grown in six-well plates were washed twice with phosphate buffered saline (PBS). The cell culture insert was then placed on top of the HaCaT cells to avoid physical contact of the different cell lines (indirect co-culture). HaCaT and THP-1 cells were stimulated with 1 μg/mL LPS (Sigma-Aldrich, St. Louis, USA) for 24 hours of co-culturing with the Transwell cell culture inserts. Additionally, 3 mL supplemented RPMI medium was added to the HaCaT monolayer (control).

### DNA microarray processing and real-time quantitative RT-PCR assays for mRNA analysis

Total RNA was extracted from cells using the High Pure RNA Isolation Kit (Roche Applied Science, Indianapolis, USA) following the manufacturer’s instructions. The quality and quantity of each RNA sample was evaluated using the RNA 6000 Nano Assay on the Agilent 2100 Bioanalyser (Agilent Technologies Inc., USA). Next, total RNA was retrotranscribed with the Transcriptor First-Strand cDNA Synthesis Kit (Roche Applied Science, Indianapolis, USA). To identify genes regulated differentially by genistein, we compared the expression levels of *ca*. 25,000 genes from genistein- and vehicle-treated keratinocytes. Gene expression data have been deposited in the NCBI’s Gene Expression Omnibus (GEO, http://www.ncbi.nlm.nih.gov/geo, GEO Series accession number GSE60971), according to the Minimum Information About a Microarray Experiment (MIAME) standards. Three biological replicates (*n*) were conducted for the microarray analysis with the use of Illumina’s Human HT-12 v4 Expression BeadChips (Illumina Inc.). The Illumina TotalPrep RNA amplification kit (Ambion) was utilized in order to amplify total input RNA. BeadChips were scanned using the Illumina BeadArray Reader and Bead Scan software (Illumina Inc.). To identify the genes that are differentially expressed between studied groups, we have applied an arbitrary threshold in the form of fold change (FC) > 1.3, and below 0.7, with the p-value 0.05 based on *t*-test comparisons. In addition, our criteria for selection of the DEGs were dictated by other considerations. A small fold change, if it co-occurred with the respective factor, would have a much larger impact than a larger fold change when the factor was not expressed. This to say that we prefer to be less stringent in detecting the DEGs, but then we put more care in extracting the important and coherent biological signals from the output (e.g., through gene set enrichment analysis [GSEA]).

In addition, we performed statistical testing for multiple comparisons by the false discovery rate (FDR) correction calculation (Benjamini-Hochberg’s test). In this, all differential expression *p*-values were FDR adjusted using the *q*-value Bioconductor package [22].

Real-time quantitative reverse transcription polymerase chain reaction (real-time qRT-PCR) was carried out with the Human Reference Gene Panel (Roche Applied Science, Indianapolis, USA), Real-Time ready Custom Panels (panel 1: config. no. 100072633; and panel 2: config. no. 100078604, Roche Applied Science, Indianapolis, USA) and the LightCycler® 480 Probes Master (Roche Applied Science, Indianapolis, USA) using the Light Cycler 480 II detection system (Roche). Expression values were normalized against three control genes *RPLP0*, *TBP*, and *YWHAZ* of constant expression level. The primers used for real-time qRT-PCR assessment of psoriatic HaCaT cell line stimulation were: *LOR* (Left: GCTCTGTCTGCGGCTACTCT; Right: AGTGACCTGCTGCGAGGA), *KRT10* (Left: TAGCAGCTTTGGTGGGAGTT; Right: CTGCCACCTCCGAAACTG), *S100A7* (Left: AAGCCTGCTGACGATGATG; Right: CGAGGTAATTTGTGCCCTTT), *S100A9* (Left: AGAAGATGCACGAGGGTGAC; Right: TGGCCACTGTGGTCTTAGG) and Universal ProbeLibrary Human *TBP* Gene Assay (Roche Applied Science, Indianapolis, USA).

### Gene Ontology and gene set enrichment studies

Gene Ontology analysis and data visualization were performed using the web tools GOrilla (http://cbl-gorilla.cs.technion.ac.il/) and lists of genes with up- and down-regulated expression separately, restricting the output to biological process and cell compartment.

GSEA was performed as previously described [23, 24] to illustrate Kyoto Encyclopedia of Genes and Genomes (KEGG)_Pathways, Biological Processes and Molecular Function enriched among genistein treated HaCaT gene sets. A nominal *p*-value < 0.01 and a FDR ≤ 0.25 were used to assess the significance of the enrichment scores. GSEA ranked list of KEGG defined pathways was created with the normalized enrichment score (NES) as the leading parameter. NES is the primary statistic for examining gene set enrichment results and can be used to compare analysis results across gene sets. Additionally, a particular pathway was considered significantly enriched if its NES had a *q*-value of an FDR-correction below or equal 25%. An FDR of 25% indicates that the result is likely to be valid 3 out of 4 times. The use of a more stringent FDR cutoff may lead to overlooking potentially significant results.

### PI3K phosphorylation analysis

In this part of the work we studied the activity of phosphatidylinositol-3-kinase (PI3K) in HaCaT cells treated with 0.05% DMSO only, stimulated with a combination of proinflammatory “cytokine mix” and treated with 100 μM genistein or stimulated with a combination of proinflammatory “cytokine mix” and treated with wortmannin (a PI3K inhibitor). Percentage of inactivated cells, activated cells (via PI3K phosphorylation), and non-expressing cells was determined for each experimental condition. HaCaT cells were seeded at a density of 4 x 10^5^ cells/well into 6-well plates in defined Keratinocyte-SFM medium (Gibco, Thermo Fisher Scientific, CA, USA) supplemented with a pituitary extract including BPE and EGF (Gibco, Thermo Fisher Scientific, CA, USA). After 24 hours, the culture medium was replaced with serum-free keratinocyte medium without growth supplements for a 16-hour incubation. For further experiments cells were pretreated with 100 μM genistein for 2 hours, and then incubated with a combination of proinflammatory “cytokine mix” (Gibco, Thermo Fisher Scientific CA, USA; Sigma-Aldrich, St. Louis, USA) for 30 minutes. The control cells were left untreated. The suspended cells were collected, and the phosphorylation of PI3K was determined by MUSE® Cell Analyzer (Merck Millipore, Germany) using a commercial kit Muse® PI3K Activation Dual Detection Kit (Merck Millipore, Germany) according to the manufacturer’s instructions. An average of 10,000 cells were analyzed for each condition. Triplicate independent experiments were conducted.

### NF-κB activation assessment

For immunostaining, rabbit monoclonal antibody NF-κB p65 (D14E12) XP® Rabbit mAb (Cell Signaling Technology, Danvers, USA) was used (1:400, incubated overnight in 4°C) with a secondary antibody, anti-rabbit IgG (H+L), F(ab')2 Fragment (Alexa Fluor® 488 Conjugate, Cell Signaling Technology, Danvers, USA) used at 1:250 (incubated 2 hours at room temperature in the dark). Rabbit serum (10%) (Sigma-Aldrich, St. Louis, USA), used as a blocking buffer, eliminated all non-specific binding of the secondary antibody. Nuclei were counterstained with 4’,6-diamidino-2-phenylindole (DAPI) (Slow Fade Diamond Antifade Mountant with DAPI, Molecular Probes, Eugene, OR, USA) for 1 hour in the dark. Images were taken using a fluorescent microscope (Leica) with x40 magnification. Cells were plated in chamber slides (Millicell EZ SLIDES, Merck Millipore, Billerica, MA, USA) in supplemented defined Keratinocyte-SFM medium (Gibco, Thermo Fisher Scientific, CA, USA). After a 24-hour incubation, cells were cultivated in serum-free keratinocyte medium without growth supplements for 16 hours. For further experiments, the cells were pretreated with 100 μM genistein for 2 hours, and then incubated with a combination of proinflammatory “cytokine mix” (Gibco, Thermo Fisher Scientific, CA, USA; Sigma-Aldrich, St. Louis, USA) for 30 minutes. The control cells were left untreated.

### Detection of reactive oxygen species (ROS) by fluorescent microscopy

To elucidate the mechanism of genistein-induced inhibition of the NF-κB nuclear translocation, we performed experiments to determine the effects of the tested isoflavone compound on intracellular reactive oxygen species (ROS) accumulation. The intracellular accumulation of ROS was monitored using CellROX® Deep Red Reagent (Molecular Probes, Thermo Fisher Scientific, CA, USA). HaCaT cells were seeded in chamber slides (Millicell EZ SLIDES, Merck Millipore, Billerica, MA, USA) in supplemented defined Keratinocyte-SFM medium (Gibco, Thermo Fisher Scientific, CA, USA). After a 24-hour incubation cells were further cultivated in serum-free keratinocyte medium without growth supplements for 16 hours. For experimental procedure cells were pretreated with 100 μM genistein or 10 mM N-acetyl-l-cysteine (NAC) for 2 hours, and then incubated with a combination of proinflammatory “cytokine mix” or TNF-α 10 ng/mL or LPS 1μg/mL (Gibco, Thermo Fisher Scientific, CA, USA; Sigma-Aldrich, St. Louis, USA) for 30 minutes. The control cells were left untreated. At the end of the treatment, cells were loaded with the CellROX® Reagent at a final concentration of 5 μM and incubated for 30 minutes at 37°C. Cells were then washed three times with PBS. Nuclei were counterstained with DAPI (Slow Fade Diamond Antifade Mountant with DAPI, Molecular Probes, Eugene, OR, USA) for 1 hour in the dark. Samples were observed under a fluorescence microscope (Leica) with x40 magnification.

### Analysis of ROS by fluorescent cell analyzer

Quantitative measurements of ROS was acquired with Muse® Oxidative Stress Kit (Merck Millipore, Germany). HaCaT cells were seeded at a density of 4 x 10^5^ cells/well into 6-well plates in defined Keratinocyte-SFM medium (Gibco, Thermo Fisher Scientific, CA, USA) supplemented with a pituitary extract including BPE and EGF (Gibco, Thermo Fisher Scientific, CA, USA). After a 24-hour incubation, cells were cultivated in serum-free keratinocyte medium without growth supplements for 16 hours. For experimental analysis cells were pretreated with 100 μM genistein or 10 mM NAC for 2 hours, and then incubated with a combination of proinflammatory “cytokine mix” or TNF-α 10 ng/mL or LPS 1μg/mL (Gibco, Thermo Fisher Scientific, CA, USA; Sigma-Aldrich, St. Louis, USA) for 30 minutes. The control cells were left untreated. Trypsinized cells were collected, and the count and percentage of cells undergoing oxidative stress based on the intracellular detection of superoxide radicals were determined by MUSE® Cell Analyzer (Merck, Millipore, Germany) using a commercial kit Muse® Oxidative Stress Kit (Merck Millipore, Germany) according to the manufacturer’s instructions. An average of 10,000 cells were analyzed for each condition. Triplicate independent experiments were conducted.

### ELISA for cytokines

The cells were seeded at the 4 x 10^5^ cells per well in 6-well-plates in defined Keratinocyte-SFM medium (Gibco, Thermo Fisher Scientific, CA, USA) supplemented with a pituitary extract including BPE and EGF (Gibco, Thermo Fisher Scientific, CA, USA). After a 24-hour incubation cells were cultivated in serum-free keratinocyte medium without growth supplements for 16 hours. Then, the cells were, respectively, pretreated with 100 μM genistein or 1 μM MTX for 2 hours and stimulated with a combination of proinflammatory “cytokine mix” or TNF-α 10 ng/mL or LPS 1μg/mL (Gibco, Thermo Fisher Scientific, CA, USA; Sigma-Aldrich, St. Louis, USA) for 24 h. The control cells were left untreated. The IL-1B, IL-8, IL-20, CCL2, and TGF-β1 levels in the cell culture supernatants were measured by enzyme-linked immunosorbent assay (ELISA) using kits purchased from EIAab Science (Wuhan, China). The optical density of each well was determined using a microplate reader (VICTOR Multilabel Plate Reader, PerkinElmer).

## Results

### Identification of genistein-responsive genes

No remarkable cytotoxicity features of genistein were observed in the range of tested conditions, while inhibition of proliferation of keratinocytes in a dose-dependent manner was detected (S1 Fig and raw data in S1 Table, S2 Table). Although a decline in proliferation by 50% was observed for keratinocytes incubated in the presence of about 20 μM genistein for more than 7 days, we decided to set the concentration of genistein at 100 μM for the following experiments, as rather short in time expositions of cells (i.e., maximally 48 hours) to the compound were carried out.

**Fig 1:**
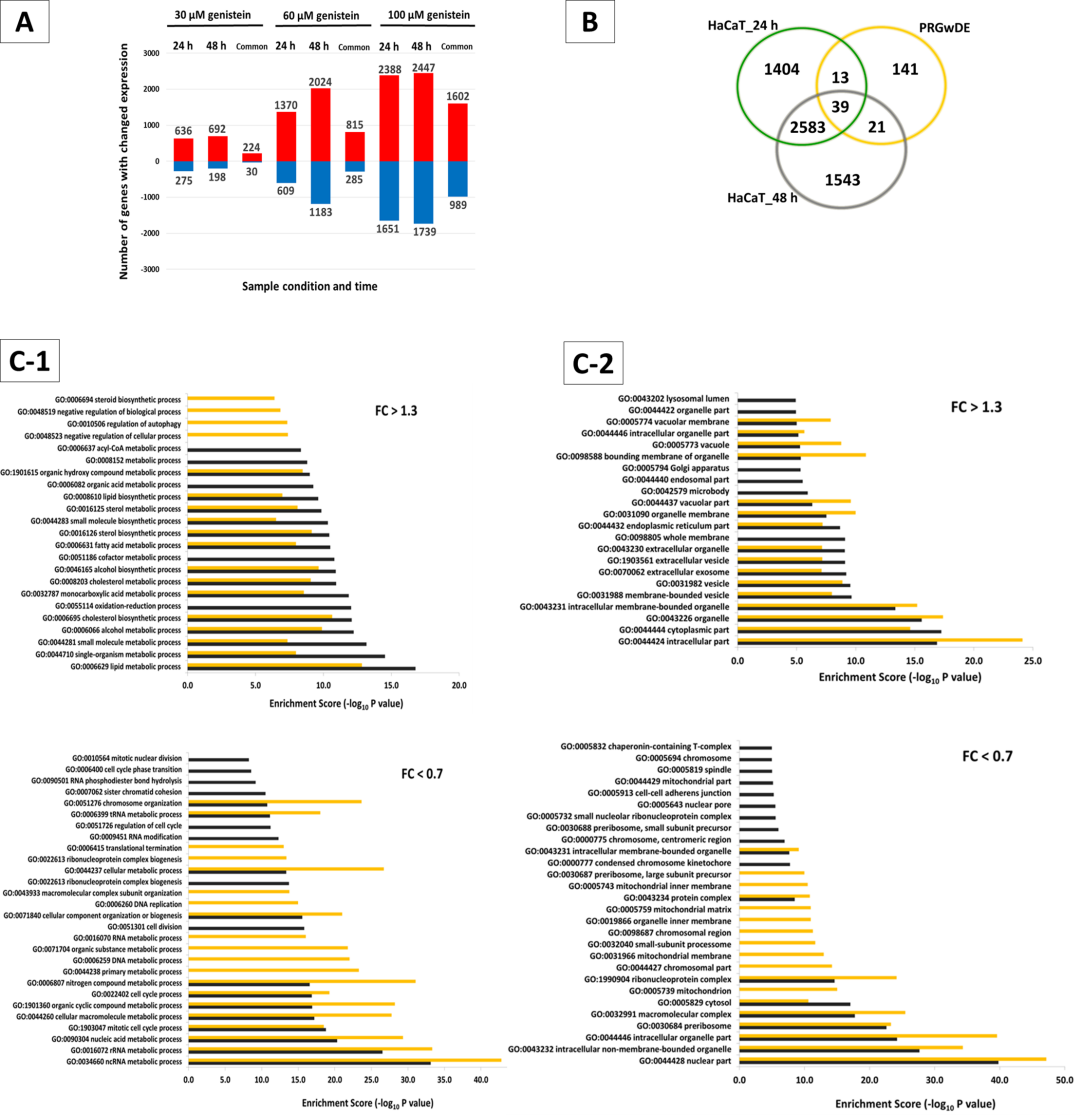
Identification of genistein-responsive genes. (A) Graphs illustrating distribution of up- (in red) and down-regulated (in blue) genes of whole HaCaT genomes after 24 and 48 hours treatment with 30, 60, and 100 μM genistein. The digits are numbers of transcripts identified as changed under studied conditions. DMSO-treated cells were used in control experiments. Significantly differentially expressed genes had a fold change of > 1.3, and below 0.7, with the *p*-value < 0.05 and *n* = 3. (B) Venn diagrams illustrating distribution of deregulated genes of whole HaCaT genomes after 24 hours (HaCaT_24 h) and 48 hours (HaCaT_48 h) of treatment with 100 μM genistein or PRGwDE compared to the respective untreated conditions. (C) GO analysis by “Biological Processes” (C-1) and “Cellular Compartment” (C-2) category of the genes with up- and down-regulated expression upon 100 μM treatment with genistein for 24 hours (black columns) and 48 hours (yellow columns) of HaCaT, with FDR ≤ 0.25, fold change > 1.3 and < 0.7, *p* < 0.01 and *n* = 3.

To identify genes regulated differentially by genistein, we compared the expression levels of *ca*. 25,000 genes from genistein- and vehicle-treated keratinocytes. Gene expression data have been deposited in the NCBI’s Gene Expression Omnibus (GEO, http://www.ncbi.nlm.nih.gov/geo, GEO Series accession number GSE60971), according to the Minimum Information About a Microarray Experiment (MIAME) standards. Testing the effects of genistein on human HaCaT transcriptome via the microarray analysis, we found that this compound induced significant dose- and time-dependent alterations in profiles of hundreds of transcripts (Fig 1A). As discovered in three independent assays, in total 4039 transcripts for 24 hours and 4186 for 48 hours handling with 100 μM genistein were affected. These changes included many psoriasis-related genes, which design a so-called psoriasis gene-expression profile (Fig 1B). In total, 214 psoriasis related-genes with differential expression (PRGwDE) were selected on the basis of the classification criteria referring to the most up-to-date scientific reports describing a list of genes with significantly differentiated activity obtained in both *in vitro* and *in vivo* analyses when studying psoriatic phenotypes, at least a couple of times stated in unrelated literature sources [1, 2, 4–6, 25–28]. Except for published psoriasis-related DEG lists, we also studied and reviewed the psoriasis associated genes database (PAGD), as well as the database of genes associated with psoriasis (dbGAP). Among those 214 PRGwDE, 39 genes (i.e. *AKR1C3*, *ALDH3A2*, *AMD1*, *C1QBP*, *CCL2*, *CCNF*, *CD47*, *CDC25A*, *CEBPD*, *EIF5*, *EIF5A*, *FOXO1*, *GJB2*, *HPSE*, *HSPA8*, *HSPE1*, *HSPH1*, *ID1*, *IL-1A*, *IL-1B*, *IL-20RB*, *IL-8*, *IRAK1*, *KPNB1*, *LAD1*, *LDLR*, *MALL*, *MAPKAPK3*, *PGAM1*, *PGD*, *PI3*, *POR*, *RNF141*, *S100A9*, *SPRR1B*, *SRM*, *TCN1*, *TCP1* and *TIMP3*) belong to a common set of 2622 genes with modulated expression in cells exposed to 100 μM genistein for 24 and 48 hours. These genes are potential therapeutic targets due to the normalization of their dysfunctional activity in psoriatic cells after exposure to genistein (including some key transcripts) and may be important for the condition. The application of Fisher's exact test gave a one-tailed probability of *p* = 0.002523, which is significant beyond the 5% level. In total, 39 out of 214 genes belonging to the 2611 DEGs modulated by genistein are a significant quantity when referred to *ca*. 25,000 cellular genes. Interestingly, taking into consideration our previous results on the use of genistein-treated HDFa fibroblasts [29] we found that 19 (i.e., *AKR1C3*, *ALDH3A2*, *AURKA*, *CCNF*, *CD47*, *CEBPD*, *EIF5*, *FOXO1*, *HMOX1*, *HSPE1*, *IL8*, *KPNA2*, *KPNB1*, *LDLR*, *MALL*, *PGD*, *POR*, *RNF141*, and *TIMP3*) of the 39 genes deregulated upon treatment with genistein and PRGwDE were common for keratinocytes and fibroblasts, both treated with 100 μM genistein for 24 hours (S2 Fig).

**Fig 2:**
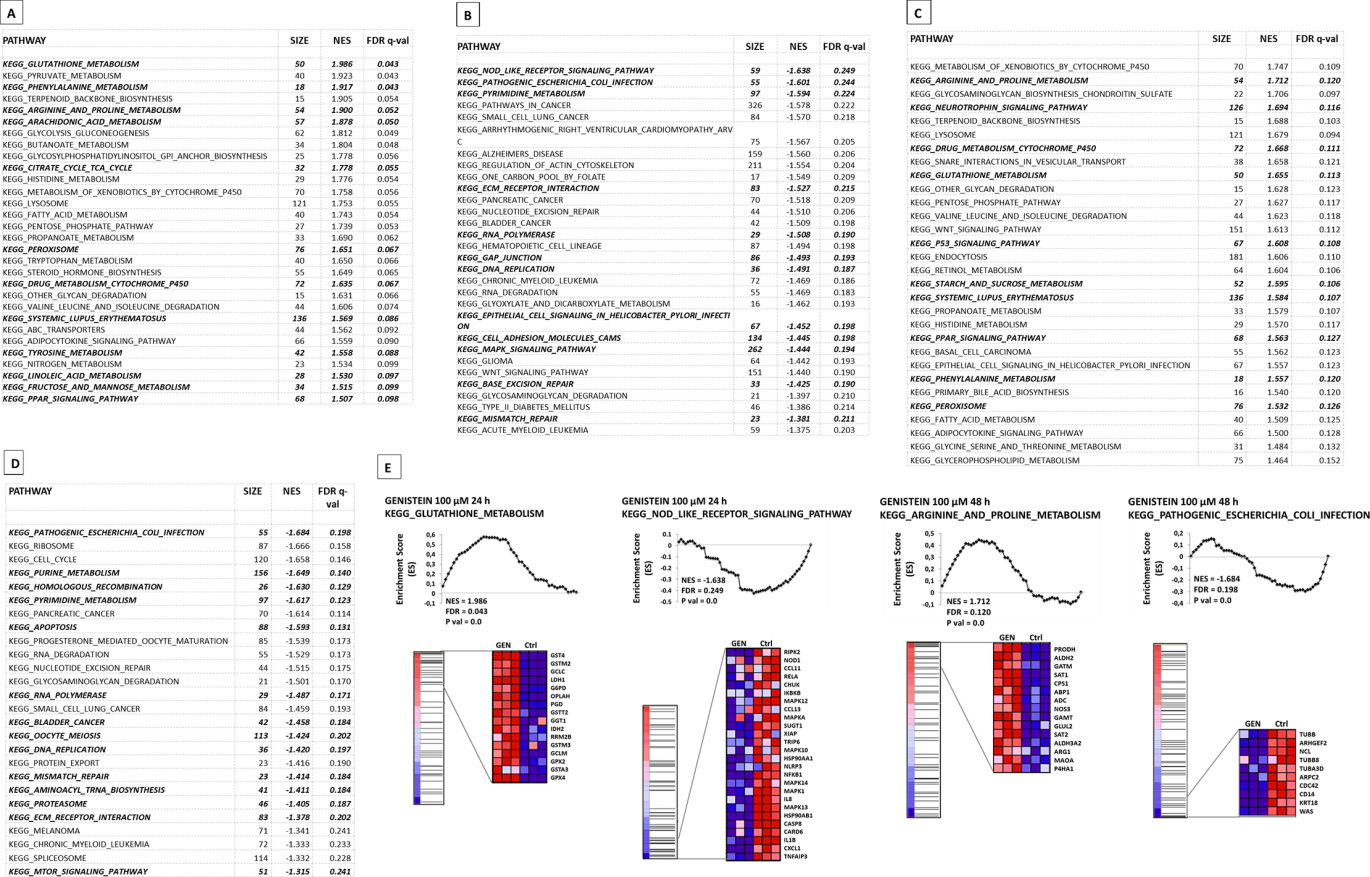
**Bench of KEGG_Pathways enriched among genes up- (A, C) and down-regulated (B, D) by genistein 24 hours (A, B) and 48 hours (D, E) treatment in HaCaT based on GSEA,** (Size, number of genes in each set; NES, normalized enrichment score; FDR *q*-val, *q*-value of false discovery rate), with gene sets of significant role in pathogenetic mechanisms responsible for the induction of psoriasis. The top 30 canonical pathways from 56 and 53 up- and down-regulated, respectively, after 24 hours and from 53 and 26 up- and down-regulated, respectively, after 48 hours treatment with genistein, enriched among the leading edge gene subsets, *p*-value < 0.01 and FDR ≤ 0.25 are illustrated. KEGG_Pathways with roles in psoriasis development are marked in bold italics. (E) GSEA analysis of mostly enriched KEGG_Pathways with roles in psoriasis development. The enrichment score is shown as a scattered black line, and the horizontal black bars next to the plot indicate the position of pathway-associated genes. Fraction of genes with essentially modulated expression is shown as GSEA-derived heat map.

Furthermore, by selecting informative genes from microarray data via Gene Ontology (GO) analysis, we recognized that tested conditions altered the expression of genes belonging to a wide range of pathways involved in “Biological processes’” and “Cellular Compartment” organization (Fig 1C). The functional annotation analyses of the genes with up-regulated expression upon treatment with genistein of HaCaT highlighted GO groups associated with lipid metabolic pathways, autophagy regulation processes and oxidation-reduction processes, while for the transcripts at the reduced level, a number of processes involved in ncRNA, rRNA, nucleic acid and cell cycle procedures were revealed (Fig 1C–1). Further, GO “Cellular Compartment” categories related to intracellular, cytoplasmic and membrane-bounded organelles were highlighted for the analysis of the up-regulated genes from the comparison between genistein-treated and control keratinocytes. In turn, the examination of genes with down-regulated expression upon treatment with genistein of HaCaT showed modulation of transcripts related to nuclear and intracellular organelle parts, pre-ribosome and mitochondrial compartments (Fig 1C–2). Accordingly, bench of KEGG_Pathways enriched among genes up- and down-regulated by 100 μM genistein treatment for 24 and 48 hours in HaCaT cells based on GSEA analysis is presented in Fig 2, sections A–D, with a significant enrichment of gene sets (marked in bold italics) with important roles in pathogenetic mechanisms responsible for the induction of psoriasis or in any other ways related to this dermatosis. In general, interpretation of the biological meaning of defining gene sets described in detail in the table in Fig 2 revealed the top 30 canonical pathways from 56 and 53 up- and down-regulated, respectively, after 24 hours and from 53 and 26 up- and down-regulated, respectively, after 48 hours treatment with genistein enriched among the leading edge gene subsets, with *p*-value < 0.01 and FDR ≤ 0.25. Within the list of up-regulated gene sets, various metabolic pathways, the peroxisome and peroxisome proliferator-activated receptor (PPAR) signaling pathway (important in psoriasis and keratinocyte homeostasis), p53 signaling pathway, and some more were found. Among the down-regulated gene sets, we detected genes belonging to a wide range of pathways involved in nucleotide-binding oligomerization domain-like (NOD-like) receptor signaling, pathogenic *Escherichia coli* infection, purine and pyrimidine metabolism, extracellular matrix (ECM) receptor interaction associated with the mutual communications between the leukocytes-extracellular matrix, mitogen-activated protein kinase (MAPK) and mechanistic target of rapamycin (MTOR) signaling pathways, and apoptosis. Fig 2, section E shows graphs presenting genes and the enrichment plots generated by GSEA analysis of mostly enriched KEGG Pathways with role in psoriasis development (i.e., KEGG_Glutathione metabolism, KEGG_NOD-like receptor signaling pathway, KEGG_Arginine and proline metabolism, and KEGG_Pathogenic Escherichia coli infection). In addition, results of annotation enrichment analysis of “Biological Processes”, “Molecular Function”, and “Cellular Compartment” terms identified in the GSEA revealed that the treatment of keratinocytes with 100 μM genistein for 24 and 48 hours significantly altered the expression of a wide range of transcripts, including psoriasis-related genes involved in numerous processes activated/deactivated in psoriasis (S3 Fig).

To provide independent validation of our microarray data and to examine in more detail the expression patterns of psoriasis-related genes involved, we used a real-time qRT-PCR approach. At this point, determination of potential candidates for endogenous control as reference genes for real-time qRT-PCR was assessed using commercially available RealTime ready Human Reference Gene Panel. Moreover, statistical analyses of the normalized gene expression data were performed in Prism (BestKeeper) (S3 Table). Among all genes, three (*RPLPO*, *TBP*, and *YWHAZ–*the first two in case of panel 1 usage, and the latter two in case of panel 2 application), were selected as references. Following confirmation of microarray results by real-time qRT-PCR, 30 from 45 tested psoriasis-associated genes belonging to real-time qRT-PCR custom panel 1 is provided in Table 1. Modulation of their activities after genistein treatment, by reducing the expression efficacy of 19 transcripts with enhanced levels in HaCaT cells and by stimulating the expression efficiency of 11 mRNAs revealing decreased activity in keratinocytes, was documented.

**Table 1:** Expression patterns of 30 psoriasis-associated genes analyzed with the use of DNA microarray and real-time qRT-PCR custom panel 1.

		Microarray		Real-time qRT-PCR	
		FC ± SD			
Gene	Gene product	24 h	48 h	24 h vs. *RPLPO*	24 h vs. *TBP*
▲ up-regulated					
*ACACB*	Acetyl-CoA Carboxylase Beta	3.4 ± 0.4	3.9 ± 0.2	3.8 ± 0.7	3.9 ± 0.5
*CAT*	Catalase	1.8 ± 0.2	1.7 ± 0.1	2.3 ± 0.4	2.8 ± 0.2
*FADS1*	Fatty Acid Desaturase 1	3.7 ± 0.2	3.6 ± 0.2	3.3 ± 0.3	4.0 ± 0.2
*FASN*	Fatty Acid Synthase	2.1 ± 0.0	2.6 ± 0.4	6.8 ± 1.3	5.7 ± 1.0
*FOXC1*	Forkhead Box C1	1.6 ± 0.2	1.9 ± 0.2	1.8 ± 0.3	2.0 ± 0.2
*FOXO1*	Forkhead Box O1	1.8 ± 0.1	2.2 ± 0.2	1.7 ± 0.1	2.1 ± 0.2
*JUN*	AP-1 Transcription Factor Subunit	1.1 ± 0.1	3.2 ± 0.1	2.9 ± 0.9	2.9 ± 0.5
*MEGF9*	Multiple EGF Like Domains 9	1.7 ± 0.3	1.5 ± 0.1	2.3 ± 0.4	2.7 ± 0.2
*MYH10*	Myosin Heavy Chain 10	1.5 ± 0.1	1.8 ± 0.0	1.5 ± 0.2	1.5 ± 0.0
*PCYOX1*	Prenylcysteine Oxidase 1	1.8 ± 0.1	1.7 ± 0.1	1.6 ± 0.2	1.7 ± 0.1
*RHOB*	Ras Homolog Family Member B	2.0 ± 0.3	2.9 ± 0.3	3.7 ± 0.8	4.2 ± 0.1
*RHOBTB3*	Rho Related BTB Domain Containing 3	2.0 ± 0.0	1.8 ± 0.1	2.3 ± 0.2	2.7 ± 0.1
*SSPN*	Sarcospan	2.7 ± 0.3	2.0 ± 0.2	5.8 ± 0.5	8.5 ± 0.8
*TIMP3*	Tissue Inhibitors of Metalloproteinases 3	1.5 ± 0.3	2.0 ± 0.3	2.2 ± 0.2	2.3 ± 0.4
*TUFT1*	Tuftelin 1	1.5 ± 0.1	1.9 ± 0.0	2.4 ± 0.3	2.6 ± 0.2
*UST*	Uronyl 2-sulfotransferase	2.3 ± 0.2	2.8 ± 0.1	2.1 ± 0.3	2.5 ± 0.1
*ZNF12*	Zinc Finger Protein 12	1.2 ± 0.1	1.7 ± 0.1	1.5 ± 0.2	1.5 ± 0.1
*ZNF483*	Zinc Finger Protein 483	2.1 ± 0.3	1.8 ± 0.4	3.2 ± 0.0	2.4 ± 0.0
*ZNF652*	Zinc Finger Protein 652	2.0 ± 0.0	1.6 ± 0.4	1.6 ± 0.0	1.8 ± 0.2
▼ down-regulated					
*CDC25A*	Cell Division Control Protein 42 Effector Protein 5	0.4 ± 0.0	0.3 ± 0.0	0.8 ± 0.2	0.7 ± 0.2
*EZH2*	Enhancer of Zeste 2 Polycomb Repressive Complex 2 Subunit	0.8 ± 0.0	0.8 ± 0.0	0.8 ± 0.1	0.9 ±0.0
*F12*	Coagulation Factor XII	0.4 ± 0.1	0.4 ± 0.0	0.2 ± 0.1	0.2 ± 0.0
*GJB2*	Gap Junction Protein Beta	0.3 ± 0.0	0.3 ± 0.0	0.3 ± 0.0	0.3 ± 0.0
*KRT6B*	Keratin 6B	0.4 ± 0.0	0.5 ± 0.0	0.4 ± 0.1	0.5 ± 0.0
*MALL*	T-cell Differentiation Protein Like	0.3 ± 0.0	0.5 ± 0.0	0.4 ± 0.1	0.5 ± 0.0
*PI3*	Peptidase Inhibitor 3	0.2 ± 0.0	0.1 ± 0.1	0.2 ± 0.1	0.3 ± 0.1
*SERPINB8*	Serpin Family B Member 8	0.4 ± 0.0	0.4 ± 0.0	0.4 ± 0.0	0.4 ± 0.0
*SRM*	Spermidine Synthase	0.5 ± 0.1	0.4 ± 0.0	0.3 ± 0.1	0.3 ± 0.0
*SYNCRIP*	Synaptotagmin Binding Cytoplasmic RNA Interacting Protein	0.3 ± 0.0	0.4 ± 0.0	0.6 ± 0.1	0.6 ± 0.1
*THBD*	Thrombomodulin	0.4 ± 0.1	0.4 ± 0.1	0.9 ± 0.2	0.9 ± 0.1

Alterations in mRNA levels of selected genes in HaCaT after 24 and 48 hours (DNA microarray) and 24 hours (real-time qRT-PCR custom panel 1) of 100 μM genistein treatment referred to 0.7 > FC > 1.3 with a *p*-value < 0.05. The microarray and real-time qRT-PCR data represent averaged values ± standard deviation (SD) from *n* ≥ 3, and denote significant differences for samples treated with genistein against non-treated samples, with respect to the reference genes *RPLPO* and *TBP* of constant expression level implemented for real-time qRT-PCR analysis. At this point determination of potential candidates for endogenous control as reference genes for real-time qRT-PCR was assessed using the commercially available RealTime ready Human Reference Gene Panel. Moreover, statistical analyses of the normalized gene expression data were performed in Prism (BestKeeper) (see S3 Table).

**Fig 3:**
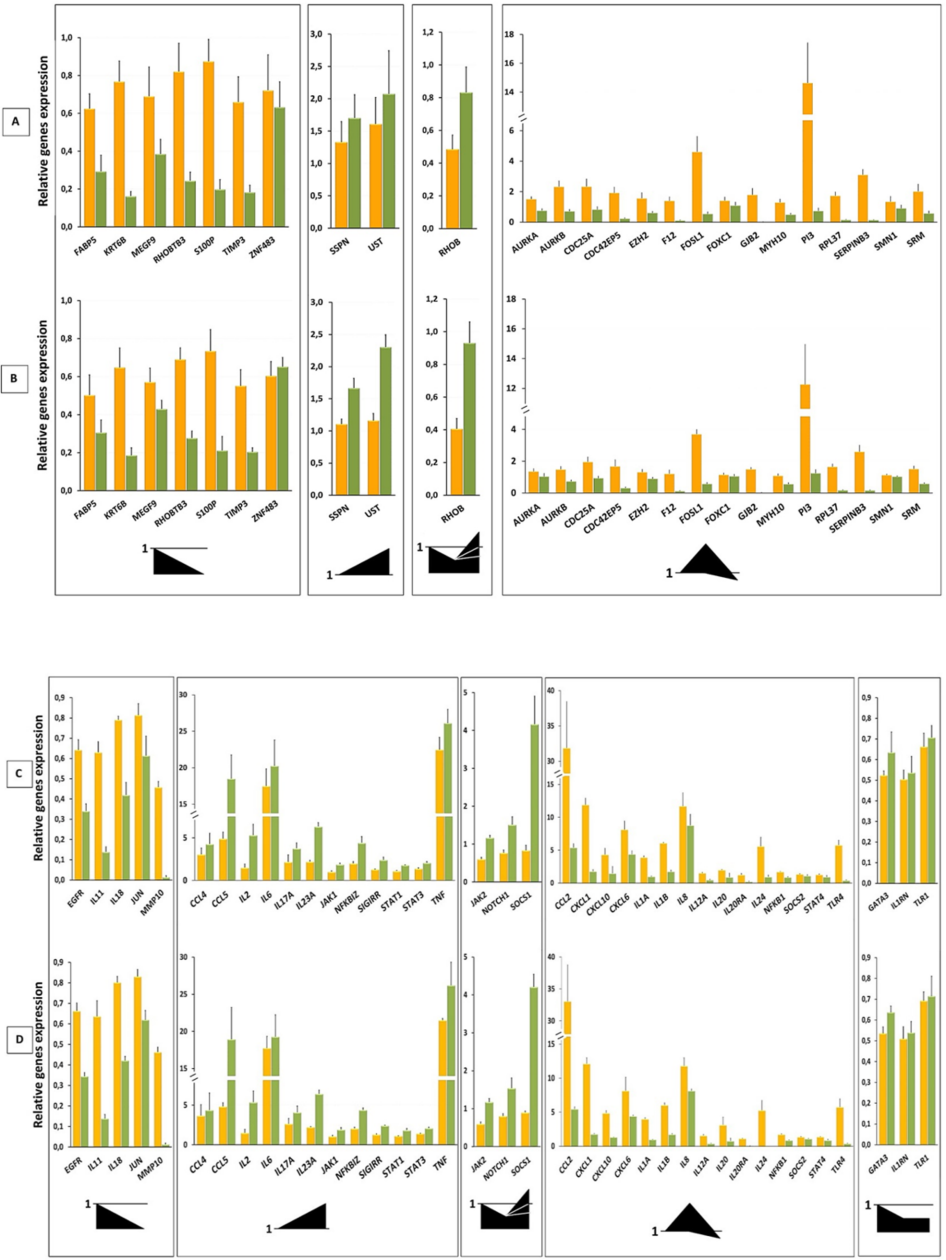
Transcriptomic profiling with the use in this case of two real-time qRT-PCR custom panels, 1 and 2, on “psoriasis-like” HaCaT cells, treated with and without genistein. Normalized to unstimulated HaCaT cells, relative real-time qRT-PCR values denoting differences for keratinocyte samples incubated with a proinflammatory “cytokine mix” (yellow bars), and keratinocyte samples incubated with a proinflammatory “cytokine mix” and treated with 100 μM genistein (green bars), with respect to the reference genes of constant expression (i.e., *RPLPO* (A) and *TBP* (B) in the case of panel 1, and *YWHAZ* (C) and *TBP* (D) in the case of panel 2). Alterations in mRNA levels of genes are referred to 0.7 > FC > 1.3 with the *p*-value < 0.05. The data represent averaged values ± standard deviation from *n* ≥ 3. The occurrence of four (A, B) or five (C, D) various profiles of gene responses is imaged for each group in the form of graphical inserts located at the bottom of the charts.

As already mentioned above, although genistein exerts potent anti-inflammatory effects, DNA microarray analysis on HaCaT cells hardly revealed modulation in activity of genes of inflammation-immune axis regulation. This in turn could be due to the applied cell culture model of normal, not mimicking or non-psoriatic keratinocytes. A 2D cell culture model of human skin that closely mimics the pathways leading to psoriatic skin formation was therefore developed and evaluated at the next phase of our work (S4 Table). Among the three different approaches tested in our study to induce a “psoriasis-like” inflammatory response in keratinocytes, we selected a method with direct treatment of the HaCaT cells with a mix of proinflammatory cytokines (IL-1A, IL-17A, IL-22), oncostatin M (OSM) and tumor necrosis factor-α (TNF-α) as the most efficient in terms of the expected characteristics. The evaluation results indicated that this assay model is able to process and provide an *in vitro* system capable of testing for a compounds ability to inhibit psoriasis-driving mechanisms (S4 Table). Thus, transcriptomic profiling with the use in this case of two real-time qRT-PCR custom panels, 1 and 2 (Real-Time ready Custom Panels for screening of in sum 90 psoriasis-related transcripts), on “psoriasis-like’” HaCaT cells, treated and untreated with genistein, was performed and determined relative to those on genistein-untreated non-psoriatic (i.e., not stimulated with a combination of proinflammatory “cytokine mix”) keratinocytes (Fig 3A–3D). The analysis revealed significant alteration in the activity of 25 of 45 genes tested with the use of panel 1, and 38 of 45 genes tested with the use of panel 2. Measurements determined by the two transcript assessment systems, panel 1 and panel 2, using endogenous references *RPLPO* (Fig 3A) and *TBP* (Fig 3B) in the case of panel 1 and using endogenous references *YWHAZ* (Fig 3C) and *TBP* (Fig 3D) in the case of panel 2, showed various clusters of gene activity. Except for a set of genes with unchanged expression for both panels (i.e., 20 transcripts of panel 1 representative of psoriasis-related genes and 7 transcripts of panel 2 representative of inflammation-immune axis regulation genes), we observed the occurrence of four main profiles of gene responses composing particular clusters, common for both panels 1 and 2. The first and second cluster, respectively, cover genes of their reduced activity or of their increased expression in response to both “cytokine mix” stimulation and proinflammatory “cytokine mix” stimulation plus genistein treatment. The third group includes genes of decreased expression after “cytokine mix” stimulation, while enhanced activity as a result of exposure to the “cytokine mix” plus genistein. The fourth cluster genes of increased activity after “cytokine mix” activation and its reduction after incubation with the “cytokine mix” plus genistein, as is depicted in the internal drawings in Fig 3. Additionally, in the case of panel 2 with inflammation-immune axis regulation genes, we detected the existence of an additional cluster, the fifth one, with genes of their reduced activity in response to “cytokine mix” stimulation, followed by maintenance at the same level of activity after incubation with the “cytokine mix” plus genistein (Fig 3C and 3D).

### No effect of genistein on PI3K activity in keratinocytes with induced NF-κB signaling

In this part of the work we studied the activity of PI3K in HaCaT cells treated with 0.05% DMSO only, stimulated with a combination of proinflammatory “cytokine mix”, stimulated with a combination of proinflammatory “cytokine mix” and treated with 100 μM genistein, or stimulated with a combination of proinflammatory ‘cytokine mix’ and treated with wortmannin (a PI3K inhibitor). Percentage of inactivated cells, activated cells (via PI3K phosphorylation), and non-expressing cells was determined for each experimental condition. The results obtained from three independent experiments revealed no significant differences in PI3K activity between cells activated with “cytokine mix” and non-activated cells (S5 Table). Additionally, keratinocytes stimulated with “cytokine mix” and treated with genistein exhibited PI3K activity at the level of non-activated cells. Statistical analysis performed by using one-way ANOVA and Tukey’s honest significant difference (HSD) showed no significant differences in cell viability relative to control cells (treated with 0.05% DMSO only). Despite this data, we found a two-fold increase in *PI3K* gene activity after stimulation with “cytokine mix”, followed by an app. 1.5-fold decrease after exposure of these cells to genistein (S5 Table).

**Fig 4:**
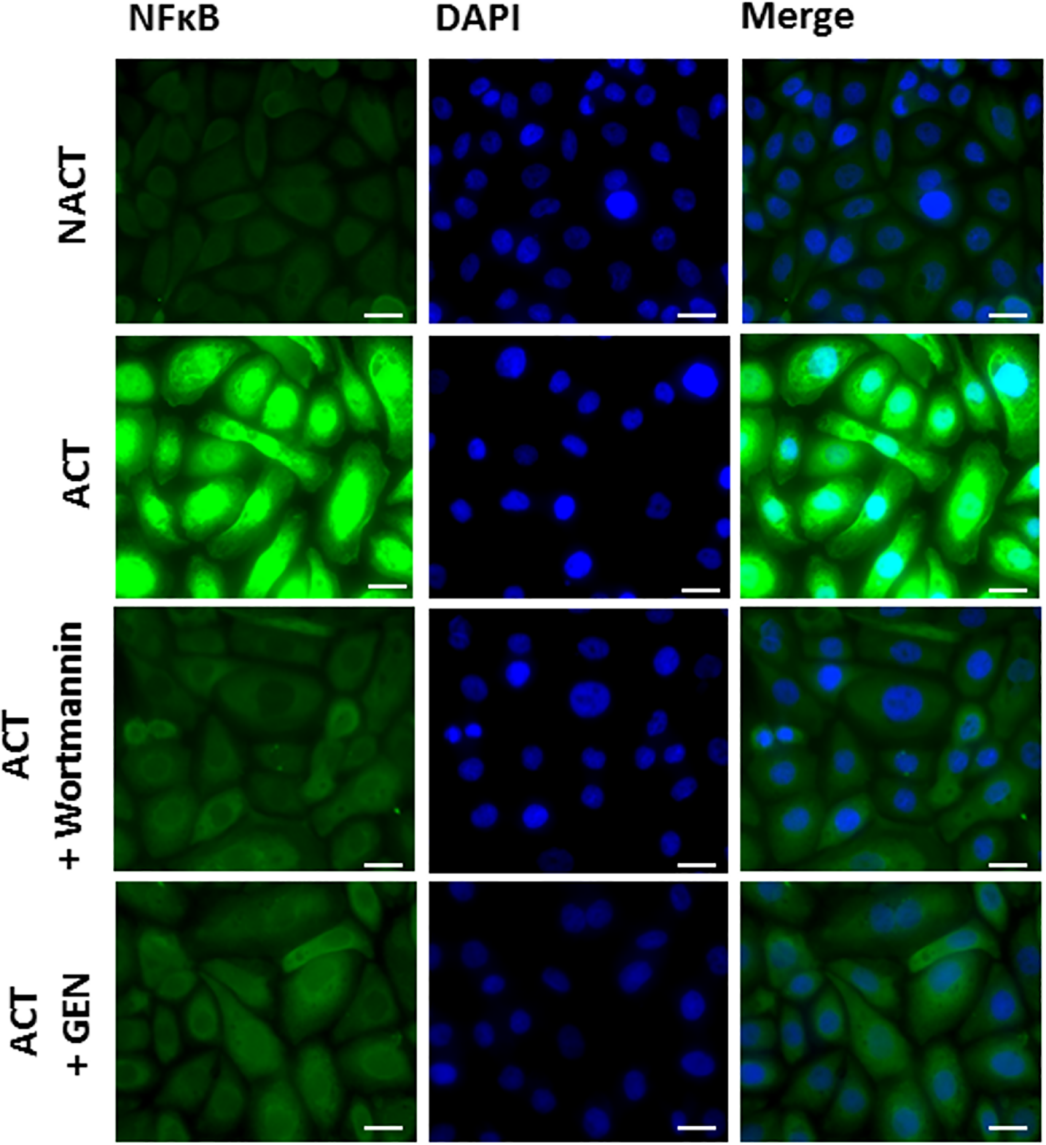
Effect of genistein on NF-κB p65 subunit induction. Keratinocytes were pretreated with or without 100 μM genistein (GEN) for 2 hours, and then incubated with a proinflammatory “cytokine mix” (ACT). DMSO-treated, unstimulated cells were used as the control (NACT). Nuclear translocation of the NF-κB p65 subunit was assessed by indirect immunofluorescence confocal microscopy using anti-p65 subunit antibodies and appropriate fluorescently tagged secondary antibodies. Nuclei were stained with 4’,6-diamidino-2-phenylindole (DAPI). Results representative of three independent experiments (with scale bars 25 μm) are shown.

Next, we studied the effect of genistein on nuclear translocation of the NF-κB p65 subunit. To accomplish this, we performed laser scanning indirect immunofluorescence confocal microscopy using an antibody to the p65 molecule. As shown in Fig 4, the immunofluorescence staining pattern showed clearly the translocation of p65 into the nucleus of human keratinocytes after activation with a cytokine cocktail. Similarly and as expected, the p65 molecule remained in the cytoplasm of stimulated keratinocytes with a combination of proinflammatory “cytokine mix” and treated with genistein. Additionally, to assess activation of the NF-κB p65 subunit following TNF-α stimulation plus genistein exposure, we examined p65 molecule nuclear translocation in keratinocytes treated with these compounds. Our results pointed to the effect of genistein on NF-κB p65 nuclear translocation when cells were activated with TNF-α alone instead of a cytokine cocktail, as the p65 molecule remained in the cytoplasm of cells (S4 Fig). Based on these results, we conclude that genistein suppresses either “cytokine mix” or TNF-α-induced nuclear factor κB p65 subunit translocation into nucleus.

### Attenuation of the level of reactive oxygen species (ROS) by genistein

**Fig 5:**
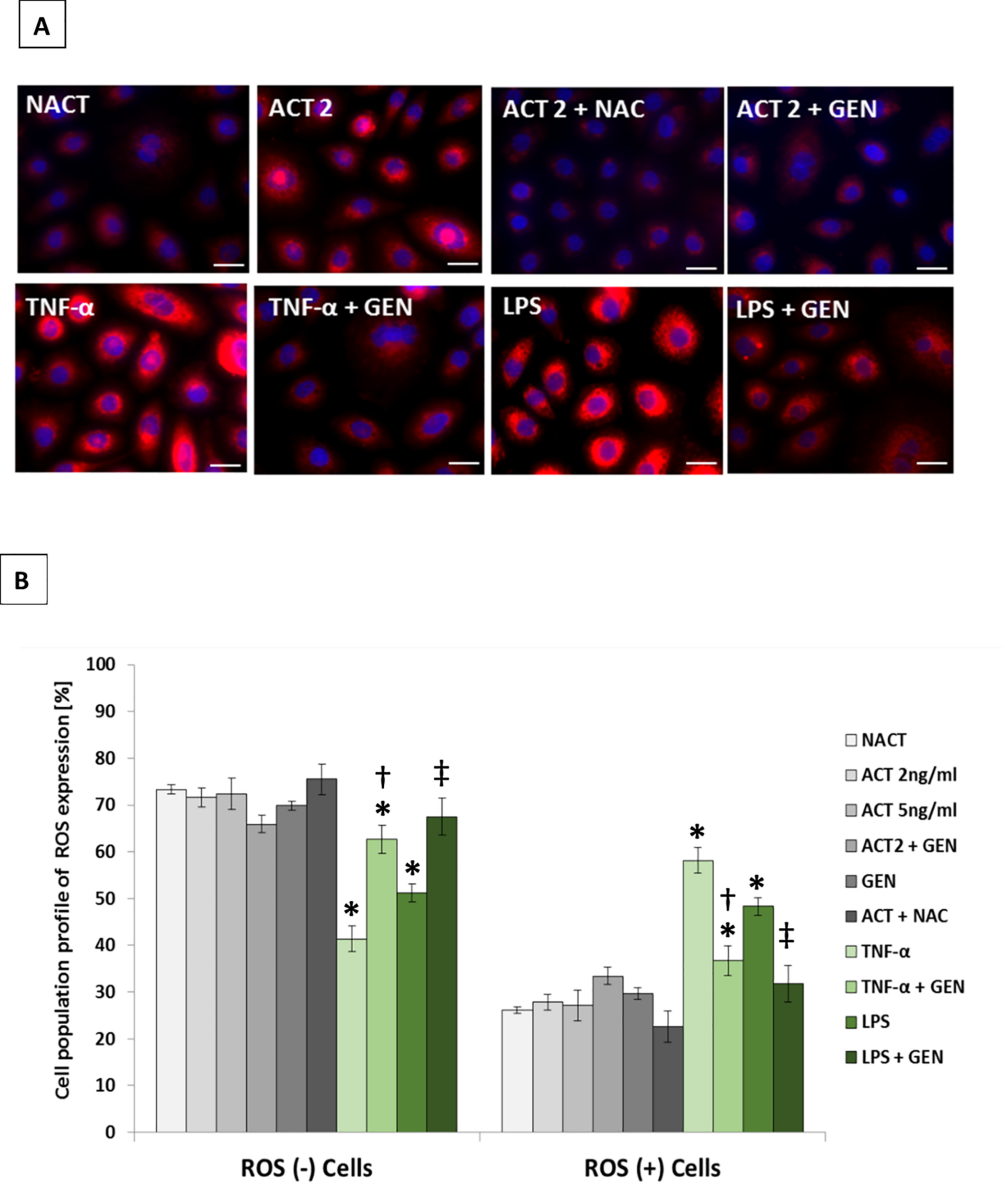
Attenuation of the level of reactive oxygen species (ROS) by genistein. (A) Keratinocytes were stimulated respectively with a proinflammatory “cytokine mix” corresponding to a concentration of 2 ng/mL (ACT 2) or 5 ng/mL (ACT 5) in each compound of the mix and incubated with 10 mM N-acetyl-cysteine (NAC) (ACT 2 + NAC), 100 μM genistein (ACT 2 + GEN), 10 ng/mL TNF-α or 1 μg/mL LPS alone, and with 10 ng/mL TNF-α or 1 μg/mL LPS incubated with 100 μM genistein (TNF-α + GEN or LPS + GEN). DMSO-treated, unstimulated cells were used as the control (NACT). Additional control was used in the form of unstimulated cells treated with 100 μM genistein (GEN). Intracellular ROS levels were examined by a CellROX Deep Red Reagent and confocal fluorescence microscopy. Nuclei were stained with 4’,6-diamidino-2-phenylindole (DAPI). Results representative of three independent experiments (with scale bars 25 μm) are shown. (B) Analysis of ROS were additionally performed by fluorescent cell analyzer. The data are presented as the means ± standard deviation (SD) from three independent experiments. Significant differences (*p* ≤ 0.05) between cell populations not expressing intracellular ROS (ROS [–]) and expressing intracellular ROS (ROS [+]) were observed for all tested conditions, except for LPS where cell numbers of ROS (-) and ROS (+) were comparable. Most important, statistically significant differences of *p* ≤ 0.05 within ROS groups are indicated with * for samples of TNF-α, TNF-α + GEN, and LPS versus NACT, with † for sample TNF-α + GEN with respect to TNF-α, while with ‡ for sample LPS + GEN referred to LPS. Statistical analysis was performed using ANOVA with Tukey’s HSD test.

To elucidate the mechanism of genistein-induced inhibition of NF-κB nuclear translocation, we performed experiments to determine the effects of the tested isoflavone compound on intracellular ROS accumulation. At first, attenuation of ROS levels by genistein was examined by confocal fluorescence microscopy (Fig 5A). In addition, significant differences (*p* ≤ 0.05) between cell populations not expressing intracellular ROS (ROS [–] cells) and expressing intracellular ROS (ROS [+] cells) were observed with the use of a fluorescent cell analyzer for all tested conditions except for LPS, where cell numbers of ROS (-) and ROS (+) cells were comparable (Fig 5B and raw data in S6 Table). Furthermore, stimulation of HaCaT keratinocytes with a combination of proinflammatory “cytokine mix” corresponding to a concentration of 2 ng/mL (ACT 2) or 5 ng/mL (ACT 5) in each compound of the mix did not increase intracellular ROS levels. Increased expression of intracellular ROS was observed only when TNF-α or LPS activation of HaCaT cells was applied, which in turn was effectively attenuated by treatment with genistein or NAC (a common scavenger of ROS) (Fig 5B and raw data in S6 Table).

### Reduction of proinflammatory cytokine production following genistein treatment

**Fig 6:**
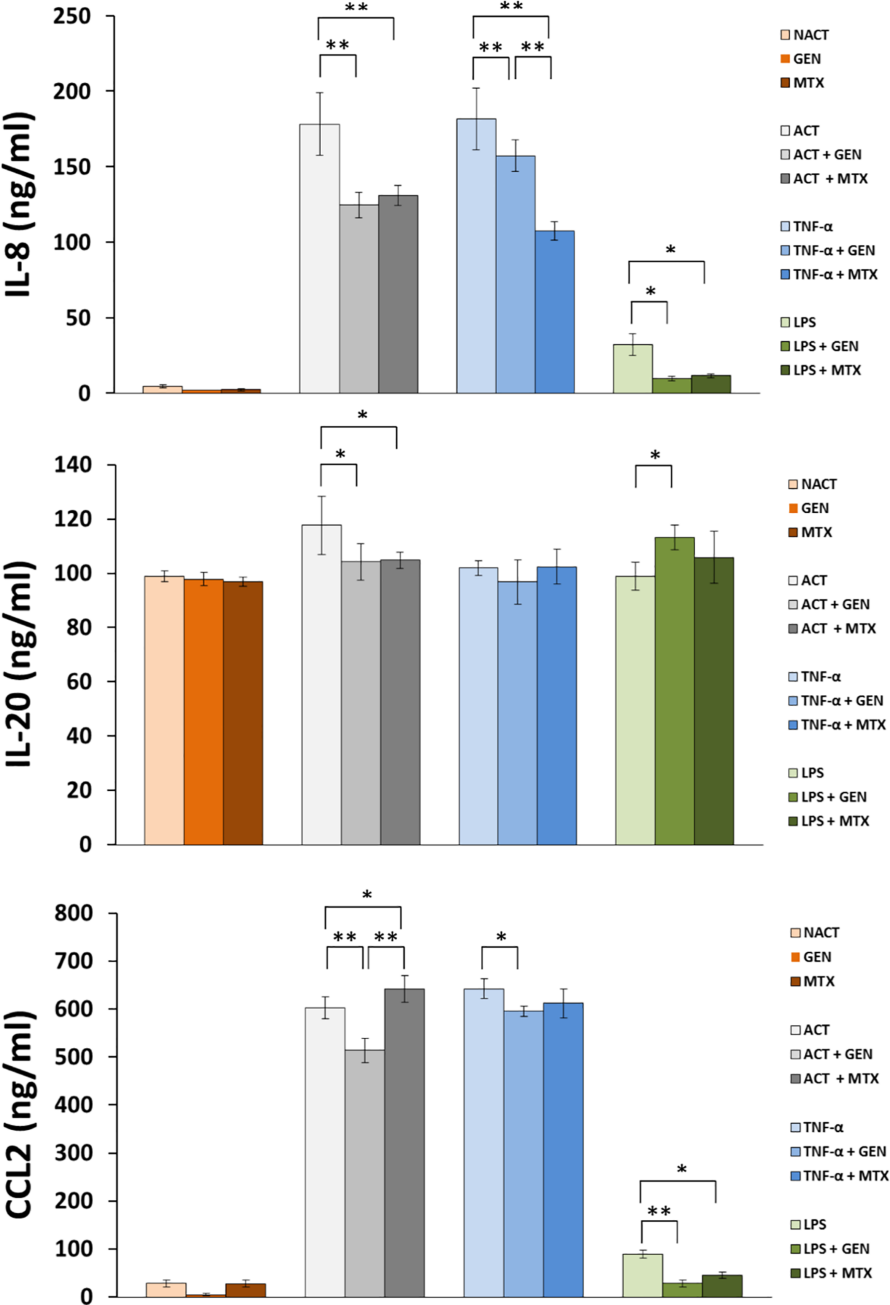
Interleukin (IL)-8, IL-20, and IL-CCL2 production determined in HaCaT cells treated correspondingly with 100 μM genistein (GEN), 1 μM methotrexate (MTX), proinflammatory “cytokine mix” at a concentration of 2 ng/mL in each compound of the mix (ACT) and incubated with 100 μM genistein (ACT + GEN) or 1 μM methotrexate (ACT + MTX), 10 ng/mL tumor necrosis factor-α (TNF-α) and incubated with 100 μM genistein (TNF-α + GEN) or 1 μM methotrexate (TNF-α + MTX), or 1 μg/mL lipopolysaccharide (LPS) alone, and incubated with 100 μM genistein (LPS + GEN) or 1 μM methotrexate (LPS + MTX). DMSO-treated, unstimulated cells were used as control (NACT). The data are presented as the means ± standard deviation (SD) from three independent experiments. Comparisons among groups were performed using a one-way ANOVA with Tukey’s HSD test and significant differences are marked with * for *p* ≤ 0.05 and with ** for *p* ≤ 0.001.

It has been well documented that overproduction of inflammatory cytokines is implicated in the pathogenesis of psoriasis. Therefore, we explored whether genistein (100 μM) could suppress expression levels of selected inflammatory proteins in keratinocytes stimulated with a combination of “cytokine mix” at a concentration of 2 ng/mL for each constituent compound or 10 ng/mL TNF-α or 1μg/mL LPS alone. In addition, 1 μM MTX (a drug well known drug diminishing cytokine production and their cellular amounts) was included in our tests [30–32]. In the course of our studies, we first found that the expression of IL-8, IL-20, and CCL2 (but not IL-1B and TGF-β1) was significantly elevated in keratinocytes regardless of the cell activation method used, except for IL-20 where neither TNF-α nor LPS stimulation influenced alterations of the level of this cytokine. Next, we showed that genistein considerably inhibits levels of IL-8, IL-20, and CCL2, but not IL-1B and TGF-β1 (Fig 6 and raw data in S7 Table). The effect of genistein was basically close to the outcome that we observed for MTX. Surprisingly, MTX, which is the drug of choice for conventional psoriasis treatment, exhibited rather weak inhibitory effects on the tested inflammatory cytokines in HaCaT cells, with the biggest impact (as much as three times more potent than genistein) only on IL-8 when cells were treated with TNF-α. On the other hand, it is also known that the results of this type of analysis may vary depending on the research model used, which in our case was HaCaT cell line (not *in vivo* material, such as serum or synovial fluid), where the effect of MTX on cytokines was conspicuous.

## Discussion

First, Ps therapies included arsenic and ammoniated mercury use in the 19^th^ Century [33, 34]. In the 1950s corticosteroids were developed as a new topical treatment [33] and were followed in the 1970s by use of MTX and psoralen ultra-violet A (PUVA) [35]. In the 1980s, Ps treatment with narrowband ultraviolet B (UVB) [36], retinoids [37], and vitamin D therapies were added [38]. From the 1990s to the present, manipulating the immune system to treat psoriasis has been explored first with cyclosporine [39] and more recently with targeted molecules–biological drugs [40]. Biologic therapies make use of specific molecules that target particular proteins implicated in immune-mediated disease. In dermatology, the approved and emerging biologic therapies work extracellularly to alter T-cell activation and differentiation, block cytokines, or eliminate pathogenic B-cells [41]. The current treatment options for psoriasis and psoriatic arthritis include TNF-α blockers and IL-12, IL-17, IL-23, T-cell and B-cell inhibitors [42]. After decades of research, there are many treatments available to help manage the symptoms of Ps, however, as yet, there is no sufficient cure. One of possible reasons might be a multi-syndromic form of the disease, which raises obstacles for one drug to focus on so many “hot spots”. Another important aspect is comorbidities, not always taken into account and hard to manage with single drug therapy. Because the disease is chronic, and essentially incurable, both acutely acting agents and those effective as long-term maintenance are needed. Natural compounds with anti-inflammatory activities are applied all over the world as alternative medicines for psoriasis because of their perceived beneficial impact on the skin. Among them are isoflavones, the most abundant phytoestrogens in soybeans (Glycine max (L.) Merr.), structurally similar to 17 beta-estradiol [43]. Soybeans and its major active compound genistein have been safely used at high levels in several Asian populations in many centuries and play a brilliant role in health promotion [44]. In this regards, we embarked on profound research on the identification of the isoflavone genistein as a potential antipsoriatic compound exerting potent anti-inflammatory effects by using an *in vitro* model of keratinocytes both normal and “psoriasis-like” types and at this phase of our work exhaustively testing genistein’s effects in a mono-treated experimental design.

The main hallmarks of Ps are abnormally proliferating and differentiating keratinocytes; therefore, each compound with the properties to reduce or normalize the increased proliferation and deregulated differentiation of psoriatic keratinocytes are strongly desired. In this report, we showed in fact that genistein significantly affected proliferation of human keratinocytes, with non-toxic activity (S1 Fig and raw data in S1 Table, S2 Table). Accordingly, the combination of the lack of cytotoxic and antiproliferative effects of genistein underlines the value of this compound and indicates that it may be a useful antipsoriatic agent.

Large scale gene expression studies of the effects of genistein on keratinocyte transcriptomes via microarray analysis revealed transcriptional changes that confirms known disease-associated pathways and highlights genomic “hot spots” for DEGs (Fig 1A). These changes included many psoriasis-related genes and were overlapped in multiple studies [1, 45]. We observed a good level of concordance between our dataset and the results of previous studies, as from 25% to 30% of dysregulated genes of keratinocytes treated with genistein for 24 and 48 hours, respectively, were present in the set of PRGwDE (Fig 1B). However, we detected the limited consensus of app. 10% microarray datasets from analyses on shared HaCaT and HDFa genes and PRGwDE transcripts, which may relate to more complex heterogeneity of subjects across studies (S2 Fig). This was not astonishing, as a study employing RNA-seq (all on psoriatic skin samples) also demonstrated the restricted consensus (with only 44% of their genes overlapping with previous microarray studies) [46]. Interestingly, our results suggested that aberrant expression of genes contributing to the progress of psoriasis could be improved by the action of genistein. Modulation of their activities, by reducing the expression efficiency of genes revealing enhanced activity in psoriatic cells and by stimulating the expression efficiency of genes revealing decreased activity in psoriatic cells, was noted. Moreover, while assembling and performing integration of our microarray data via enrichment, clustering, and correlation analyses using specific bioinformatics tools dedicated to global perspective studies, we observed a significant enrichment of functions and compartments involved in epidermal homeostasis, but also pathways related to psoriasis condition (Figs 1C and 2; S3 Fig). We detected clear modulation of psoriasis-associated transcripts levels after genistein treatment, however at this time we hardly revealed pathways related to immunity and inflammatory activation. Exceptions were the pathways of the NOD-like receptor (NLR) signaling regulation (Fig 2B and 2E) and Pathogenic Escherichia coli infection (Fig 2B, 2D and 2E), both comprising genes down-regulated by genistein. These results are interesting in respect to the data reported by Tervaniemi’s group [47], where the NLR signaling pathway, belonging to endogenous cellular stress signals and pathogens recognized via pattern recognition receptors (PRRs), with highly up-regulated transcripts was highlighted in psoriatic skin lesions. Besides, KEGG pathway analyses performed in this work identified up-regulated genes that were enriched in such function and cellular components as the lysosome (Fig 2A and 2C; I and K sections in S3 Fig). Cellular compartment terms, such as vesicle, endoplasmic reticulum, vacuole, Golgi apparatus, and lysosomal lumen, were among the most significant clusters as well (Fig 1C–2). This seems to be intriguing in light of our earlier research concerning the modulation of expression of lysosomal metabolism by flavonoids, among them the phytoestrogen genistein [29, 48]. On the other hand, one should mention that of all the components of the cell, the lysosome is an obvious candidate for a role in the inflammatory process [49]. What is more, modulation of the inflammatory response via lysosomal signaling and inflammasome-related pathways was detected in human autoimmune-mediated inflammatory diseases [50], among them in psoriasis (one of the most representative inflammatory skin disorder) [47, 51].

Both microarray and real-time qRT-PCR analyses indicated that genistein influences the expression of numerous genes linked to psoriasis (Table 1). The altered expression signatures point to stimulation of mainly the AMP-activated protein kinase (AMPK) signaling pathway (known to limit inflammation) [52–54]), Fatty acid metabolism, Forkhead box O (FoxO) signaling pathway, Tight junction and Longevity regulating pathway, while to inhibition of the Cell division cycle and Metabolic signaling pathways as important targets for genistein (a candidate for psoriasis-inhibiting agent).

Despite the many controversies about the use of proper epidermal keratinocytes for *in vitro* studies relevant to Ps, most often HaCaT cells (with mutant p53 and loss of p16ink4a) have been employed as cellular models to investigate hyperproliferative skin diseases such as Ps and to evaluate the antipsoriatic activities of tested molecules [12–16, 55]. HaCaT are often used for psoriasis *in vitro* experiments instead of primary normal human epidermal keratinocytes (NHEK) since the susceptibility of this cell line to treatment may change with the increasing number of passages (whereas HaCaT cells provides an almost unlimited supply of identical cells, assuring high reproducibility) [56]. Therefore, in this study a genistein-treated human immortalized keratinocyte cell line HaCaT was utilized. In addition, for the development of a somehow more appropriate model of human skin that closely mimics the processes leading to psoriatic lesion formation, we evaluated the use of stimulated keratinocytes. As a result, we found an approach with direct treatment of the keratinocytes with a “cytokine mix” as the closest of the expected characteristics. The transcriptomic profiling with the use of real-time qRT-PCR on “psoriasis-like” HaCaT cells treated with and without genistein (determined via measurements with the use of panel 1) revealed significant alteration in the activity of most of the tested psoriasis-related genes (Fig 3A and 3B). Moreover, expression profiling of selected genes of inflammation-immune axis regulation (determined via measurements with the use of panel 2) indicated important gene activity changes (Fig 3C and 3D). Interestingly, part of the genes from these collections depicted in Fig 3A–3D were present among the transcripts regulated in genistein-treated HaCaT included in Table 1.

To characterize further the mechanism underlying the anti-inflammatory effects of genistein, we assessed the role of NF-κB signaling cascades in the “cytokine mix” and TNF-α-mediated inflammatory responses present in keratinocytes exposed to the tested isoflavone. Translocation of the NF-κB p65 subunit into the nucleus is an important step for its transcriptional activity, which in turn seems to be mediated by PI3K signaling [57]. Previous work has reported the importance of the PI3K pathway as an important regulator of growth and inflammation in inflammation-mediated diseases such as psoriasis [58]. Moreover, studies indicated that genistein, alone or even in combination with various pharmaceutical agents (e.g., selected non-steroidal anti-inflammatory drugs), inhibits EGF receptor kinase and its downstream effector PI3K, which in turn might also be implicated in NF-κB transcriptional activation [59, 60]. Thus, to test these correlations, we primarily examined the activity of PI3K. Our experiments revealed no statistically significant differences in the activity of this kinase in keratinocytes stimulated with the cytokine cocktail versus the unstimulated one, similarly with no effect of genistein on this phenomenon (S5 Table). Interestingly, at the same time, we found modulation of *PI3K* gene expression in such conditions (S5 Table). Furthermore, genistein prevented “cytokine mix” as well as TNF-α-induced NF-κB translocation (Fig 4; S4 Fig). Thus, we conclude at lack of PI3K pathway involvement in NF-κB activation in our experimental design, which is not surprising, as it seems to be a cell- and tissue-specific event [61]. Our results regarding intracellular ROS accumulation showed that stimulation of keratinocytes with TNF-α and LPS, but surprisingly not with the “cytokine mix”, increases ROS levels, which on the other hand was effectively reduced by genistein (Fig 5). These data indicated that the tested isoflavone could attenuate TNF-α- and LPS-induced inflammatory responses in HaCaT by suppressing ROS activation. This is consistent with the antioxidant properties of genistein and reports of others, where this agent has been shown to protect cells against ROS by scavenging free radicals, enhancing activity of antioxidant enzymes and reducing production of hydrogen peroxide [62]. Our results are also corroborated by the research report of Young’s group, which has documented that ROS are involved in TNF-α-mediated signaling pathways associated with inflammatory skin disease such as psoriasis via TNF-α-dependent NF-κB activation [61]. After all, to further investigate the anti-inflammatory activity of genistein, we examined the expression of selected inflammatory mediators once the cells exposed or not exposed to genistein were stimulated either with the cytokine cocktail, TNF-α, or LPS (Fig 6 and raw data in S7 Table). We found that, regardless of the type of cell activation used, the levels of three (IL-8, IL-20 and CCL2) out of five cytokines tested were decreased in the keratinocyte supernatant in response to the isoflavone. It is worth emphasizing, too, that the same trend (i.e., first an increased expression in response to stimulation, followed by reduction after genistein treatment) was observed in both RNA and protein levels of these three cytokines (Figs 3C [the forth cluster] and 6). Based on these results, we conclude that genistein may suppress inflammatory cytokine production, at least partly, by inhibiting the ROS/NF-κB pathway in activated HaCaT cells. We, thus, hypothesize that genistein attenuates ROS-mediated NF-κB activation and subsequent inflammatory cytokine production in “psoriasis-like” keratinocytes. These data provide new insight into the anti-inflammatory mechanism and antipsoriatic activity of genistein, giving scientific support for its use in the treatment of Ps.

Our work exploits and expands on recent breakthroughs in the understanding of cellular cross-talk to develop novel therapeutic approaches based on the use of the isoflavone genistein to treat complex diseases such as psoriasis. It is believed that investigations presented in this report can provide new information regarding the molecular mechanism of the action of genistein modulating the activity of genes deregulated in the cells of people suffering from psoriatic skin disease, at the same time significantly affecting the signaling pathways in such cells. Understanding of these issues may result in serious progress in therapeutic approaches, where, as it is nowadays increasingly observed, combination therapy rather than monotherapy are more effective. Indeed, monotherapy with systemic agents is effective for many patients with Ps; however, some of them require combination approaches. Perhaps the use of genistein in such therapy with, for example, biologic drugs may have an even more beneficial outcome than being used alone. Main aspects in considering a switch from monotherapy to combination treatment are less cumulative and/or acute toxicity, fewer side effects, and obviously improved therapeutic outcomes for the latter. The research on the identification of the isoflavone genistein as a potential antipsoriatic compound exerting potent anti-inflammatory effects, together with anti-Ps systemic drugs with complementary activities may be worth the attention. Because of the safety of genistein [44], its use might be recommended as adjuvant together with other agents such as biological drugs in psoriasis management, especially while handling patients resistant to treatment.

In this study, we report the range of knowledge gained with the *in vitro* cell culture in form of keratinocytes that closely mimic the psoriatic state and on genistein used alone. The obtained results are intended to be utilized in the next phase of research involving animal models, and also the combination approach of the isoflavone genistein and selected systemic drugs. Because investigations of other groups supported our commentary on the potential administration of soybeans as a potential weapon in the armamentarium against psoriasis, it is believed that this paper should serve to encourage us and other researchers to conduct further studies on this subject.

## Supporting information

S1 Fig Effect of genistein on the viability of HaCaT measured by the level of MTT incorporated into cells. Keratinocytes were treated with different concentrations of genistein for 24 hours, 48 hours (cytotoxicity assay), and 7 days (proliferation assay); afterward the percentage of cell survival was determined. Results are expressed as mean values of three experiments with error bars indicating standard deviation.
(TIF)

S2 Fig Venn diagrams summarizing the number of deregulated genes of whole genome of HaCaT, HDFa treated with 100 μM genistein for 24 hours or PRGwDE compared to the respective untreated conditions. (TIF)

S3 Fig Results of annotation enrichment analysis. Terms identified in the GSEA for correlated genes in the categories: “Biological Processes”, “Molecular Functions” and “Cell Compartments” with up to 30 gene sets, enriched among those up- (A, C, E, G, I, K) and down-regulated (B, D, F, H, J, L) by 100 μM genistein 24 hours (A, B, E, F, I, J) and 48 hours (C, D, G, H, K, L) after treatment in HaCaT cells (Size, number of genes in each set; NES, normalized enrichment score; FDR *q*-val, *q*-value of false discovery rate). GSEA was performed using the microarray data to design a so-called psoriasis gene expression profile between keratinocytes treated with or without genistein. Tables A–D illustrate up to 30 top “Biological Processes” from 83 up-regulated and 27 down-regulated genes, after 24 hours of treatment with genistein and from 113 up-regulated and 77 down-regulated genes, after 48 hours of treatment with genistein. Tables E–H illustrate the top 30 “Molecular Functions” from 77 up-regulated and 168 down-regulated genes, after 24 hours of treatment and from 160 up-regulated and 90 down-regulated genes, respectively, after 48 hours of treatment with genistein. Tables I–L illustrate the top 30 “Cell Compartments” from 56 up-regulated and 136 down-regulated, after 24 hours of treatment and from 112 up-regulated and 111 down-regulated, respectively, after 48 hours of treatment with genistein. All were enriched among the leading edge gene subsets, with *p*-value < 0.01 and FDR ≤ 0.25.
(PDF)

S4 Fig Effect of genistein on NF-κB p65 subunit induction. Keratinocytes were pretreated with or without 100 μM genistein (GEN) for 2 hours, and then incubated with a proinflammatory “cytokine mix” (ACT) or only TNF-α (10 ng/mL) (TNF-α + GEN), for 30 minutes. Only DMSO-treated, unstimulated cells were used as control (NACT). Nuclear translocation of the NF-κB p65 subunit was assessed by indirect immunofluorescence confocal microscopy using anti-p65 subunit antibodies and appropriate fluorescently tagged secondary antibodies. Nuclei were stained with 4’,6-diamidino-2-phenylindole (DAPI). Results representative of three independent experiments (with scale bars 100 μm) are shown.
(TIF)

S1 Table (Raw data of S1 Fig) Effect of genistein on the viability of HaCaT measured by the level of MTT incorporated into cells. Keratinocytes were treated with different concentrations of genistein (GEN) for 24 hours, 48 hours (cytotoxicity assay), and 7 days (proliferation assay); afterward the percentage of cell survival was determined. Results are expressed as mean values of three experiments with error bars indicating standard deviation.
(DOCX)

S2 Table LC (cytotoxicity assay) and IC (proliferation assay) index values of genistein in keratinocyte-based assay. Cytotoxicity is expressed as LC25, 50 or 75 (i.e., concentration of the tested drug [μM]) that is lethal to 25%, 50%, or 75% of HaCaT cells, respectively, in a culture exposed to the drug for 24 and 48 hours. Antiproliferative activity is expressed as IC25, 50 or 75 (i.e., concentration of the tested drug [μM]) that causes 25%, 50%, or 75% inhibition of keratinocyte proliferation, respectively, in a culture exposed to the drug for 7 days.
(DOCX)

S3 Table Descriptive statistics of 10 candidate housekeeping genes (HKG) based on their crossing point (CP) values. Abbreviations: n: number of samples; GM [CP]: the geometric mean of CP; AM [CP]: the arithmetic mean of CP; Min [CP] and Max [CP]: the extreme values of CP; SD [± CP]: the standard deviation of the CP; CV [% CP]: the coefficient of variance expressed as a percentage on the CP level; Min [x-fold] and Max [x-fold]: the extreme values of expression levels expressed as an absolute x-fold over- or under-regulation coefficient; SD [± x-fold]: standard deviation of the absolute regulation coefficients; coeff. of corr. [r]: coefficient of correlation; coeff. of det. [r^2^]: coefficient of determination.
(DOCX)

S4 Table Expression patterns of keratinocyte differentiation markers *KRT10* and *LOR*, and inflammation markers *S100A7* and *S100A9*. The analysis was made via real-time qRT-PCR in response to various tested “psoriasis-like” activation processes: treatment with the proinflammatory “cytokine mix” (2 ng/mL of IL-1A, IL-17A, IL-22, OSM and TNF-α), co-culture of HaCaT and THP-1, with or without addition of 1 μg/mL lipopolysaccharide (LPS) for 24 hours. mRNA expression levels for the four marker genes were normalized using *TBP* housekeeping reference and expressed as the fold change for stimulated vs. unstimulated cells.
(DOCX)

S5 Table Activity of PI3 kinase and *PI3K* gene in HaCaT. Keratinocytes were treated with 0.05% DMSO only (NACT), stimulated with a combination of proinflammatory “cytokine mix” (ACT), stimulated with a combination of proinflammatory “cytokine mix” and treated with 100 μM genistein (GEN), or stimulated with a combination of proinflammatory “cytokine mix” and treated with wortmannin (a PI3K inhibitor) (WORT).
(DOCX)

S6 Table (Raw data of Fig 5(B)) attenuation of the level of reactive oxygen species (ROS) by genistein. (B) Analysis of ROS were additionally performed by fluorescent cell analyzer. The data are presented as the means ± standard deviation (SD) from three independent experiments. Significant differences (*p* ≤ 0.05) between cell populations not expressing intracellular ROS (ROS [–]) and expressing intracellular ROS (ROS [+]) were observed for all tested conditions, except for LPS where cell numbers of ROS (-) and ROS (+) were comparable. Statistical analysis was performed using ANOVA with Tukey’s HSD test.
(DOCX)

S7 Table (Raw data of Fig 6) interleukin (IL)-8, IL-20, and CCL2 production determined in HaCaT cells treated correspondingly with 100 μM genistein (GEN), 1 μM methotrexate (MTX), proinflammatory “cytokine mix” at a concentration of 2 ng/mL in each compound of the mix (ACT) and incubated with 100 μM genistein (ACT + GEN) or 1 μM methotrexate (ACT + MTX), 10 ng/mL tumor necrosis factor-α (TNF-α) and incubated with 100 μM genistein (TNF-α + GEN) or 1 μM methotrexate (TNF-α + MTX), or 1 μg/mL lipopolysaccharide (LPS) alone, and incubated with 100 μM genistein (LPS + GEN) or 1 μM methotrexate (LPS + MTX). DMSO-treated, unstimulated cells were used as control (NACT). The data are presented as the means ± standard deviation (SD) from three independent experiments. Comparisons among groups were performed using a one-way ANOVA with Tukey’s HSD test.
(DOCX)
