# Supplementary figures and images for: Molecular action of isoflavone genistein in the human epithelial cell line HaCaT
Source: PLoS One. 2018 Feb 14;13(2):e0192297. doi: 10.1371/journal.pone.0192297 (PMC5812592; doi:10.1371/journal.pone.0192297)

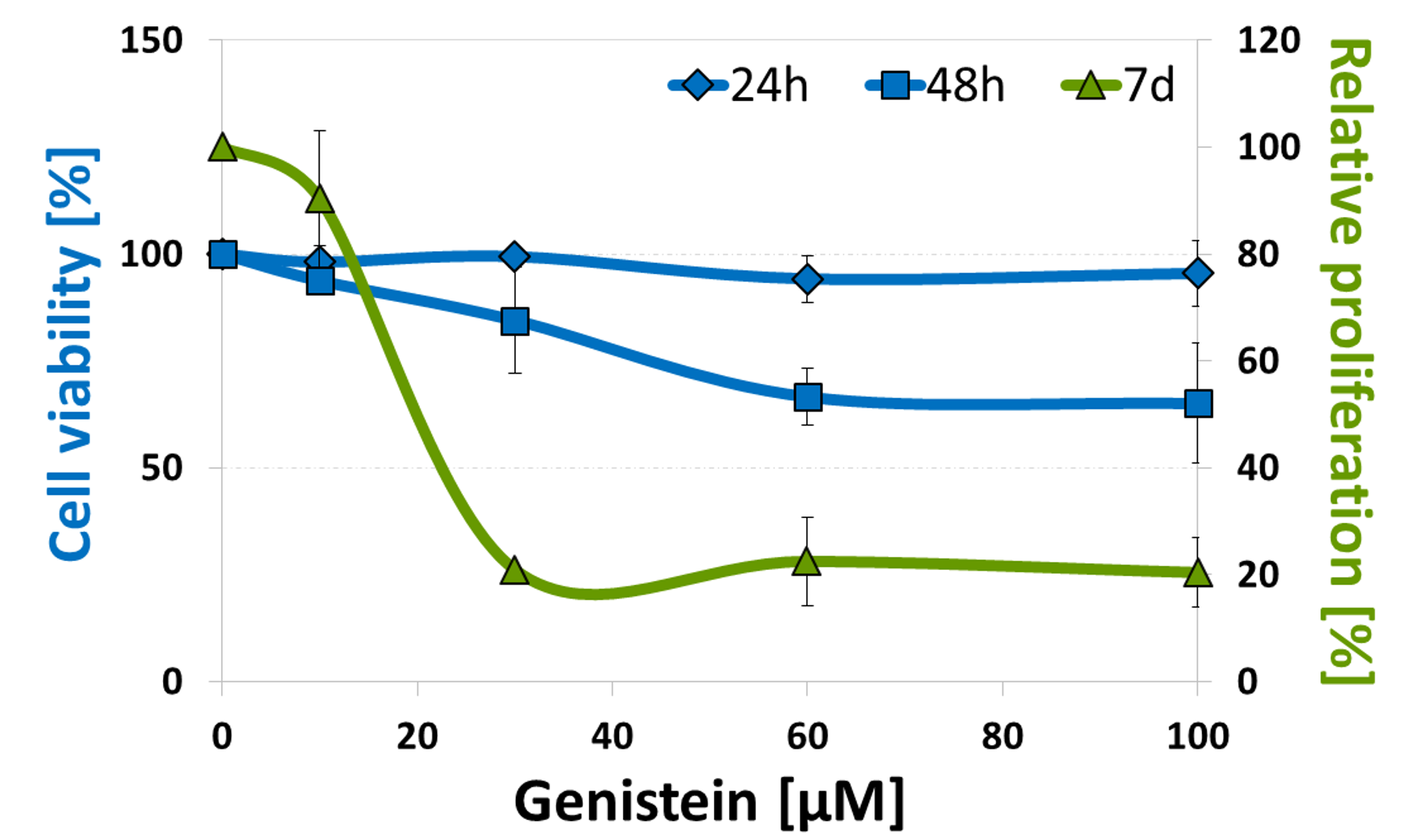

Supplement: S1 Fig — Keratinocytes were treated with different concentrations of genistein for 24 hours, 48 hours (cytotoxicity assay), and 7 days (proliferation assay); afterward the percentage of cell survival was determined. Results are expressed as mean values of three experiments with error bars indicating standard deviation. (TIF) [file pone.0192297.s001.tif]

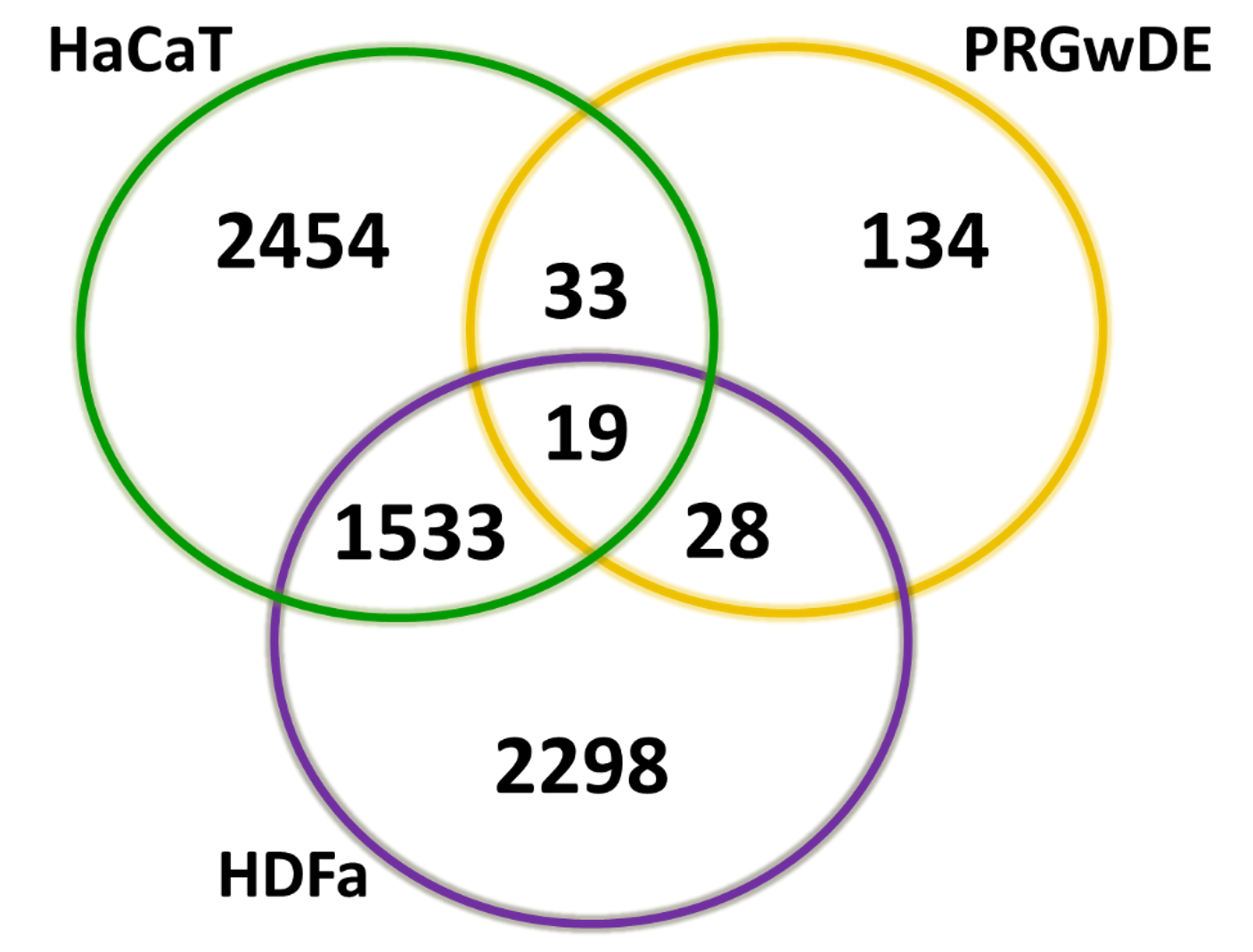

Supplement: S2 Fig — (TIF) [file pone.0192297.s002.tif]

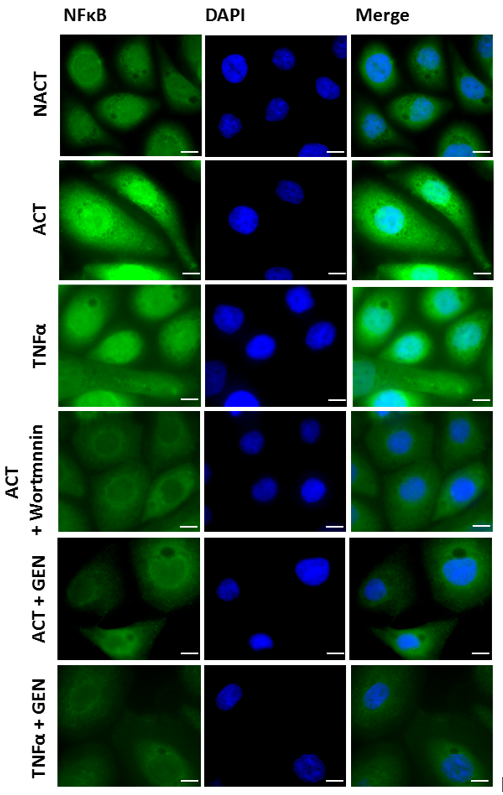

Supplement: S4 Fig — Keratinocytes were pretreated with or without 100 μM genistein (GEN) for 2 hours, and then incubated with a proinflammatory “cytokine mix” (ACT) or only TNF-α (10 ng/mL) (TNF-α + GEN), for 30 minutes. Only DMSO-treated, unstimulated cells were used as control (NACT). Nuclear translocation of the NF-κB p65 subunit was assessed by indirect immunofluorescence confocal microscopy using anti-p65 subunit antibodies and appropriate fluorescently tagged secondary antibodies. Nuclei were stained with 4’,6-diamidino-2-phenylindole (DAPI). Results representative of three independent experiments (with scale bars 100 μm) are shown. (TIF) [file pone.0192297.s004.tif]
