# Supplementary material for: Molecular action of isoflavone genistein in the human epithelial cell line HaCaT
Source: PLoS One. 2018 Feb 14;13(2):e0192297. doi: 10.1371/journal.pone.0192297 (PMC5812592; doi:10.1371/journal.pone.0192297)
Supplement: S3 Fig — Terms identified in the GSEA for correlated genes in the categories: “Biological Processes”, “Molecular Functions” and “Cell Compartments” with up to 30 gene sets, enriched among those up- (A, C, E, G, I, K) and down-regulated (B, D, F, H, J, L) by 100 μM genistein 24 hours (A, B, E, F, I, J) and 48 hours (C, D, G, H, K, L) after treatment in HaCaT cells (Size, number of genes in each set; NES, normalized enrichment score; FDR q-val, q-value of false discovery rate). GSEA was performed using the microarray data to design a so-called psoriasis gene expression profile between keratinocytes treated with or without genistein. Tables A–D illustrate up to 30 top “Biological Processes” from 83 up-regulated and 27 down-regulated genes, after 24 hours of treatment with genistein and from 113 up-regulated and 77 down-regulated genes, after 48 hours of treatment with genistein. Tables E–H illustrate the top 30 “Molecular Functions” from 77 up-regulated and 168 down-regulated genes, after 24 hours of treatment and from 160 up-regulated and 90 down-regulated genes, respectively, after 48 hours of treatment with genistein. Tables I–L illustrate the top 30 “Cell Compartments” from 56 up-regulated and 136 down-regulated, after 24 hours of treatment and from 112 up-regulated and 111 down-regulated, respectively, after 48 hours of treatment with genistein. All were enriched among the leading edge gene subsets, with p-value < 0.01 and FDR ≤ 0.25. (PDF) [file pone.0192297.s003.pdf]

A

BIOLOGICAL PROCESS	SIZE	NES	FDR *q*-val
**Response to stimulus**			
CELLULAR_RESPONSE_TO_STIMULUS	19	2.220	0.054
RESPONSE_TO_EXTRACELLULAR_STIMULUS	33	2.162	0.054
RESPONSE_TO_NUTRIENT_LEVELS	29	2.115	0.054
**Lipid metabolic process**			
ORGANIC_ACID_METABOLIC_PROCESS	177	1.856	0.077
CARBOXYLIC_ACID_METABOLIC_PROCESS	175	1.850	0.073
LIPID_METABOLIC_PROCESS	318	1.838	0.069
CELLULAR_LIPID_METABOLIC_PROCESS	250	1.776	0.076
FATTY_ACID_METABOLIC_PROCESS	61	1.722	0.091
LIPID_BIOSYNTHETIC_PROCESS	95	1.690	0.107
ALCOHOL_METABOLIC_PROCESS	87	1.890	0.066
ORGANIC_ACID_TRANSPORT	42	1.689	0.105
**Others**			
ELECTRON_TRANSPORT_GO_0006118	51	1.975	0.054
GENERATION_OF_PRECURSOR_METABOLITES_AND_ENERGY	122	1.838	0.071
GLUCOSE_METABOLIC_PROCESS	28	1.776	0.074

B

BIOLOGICAL PROCESS	SIZE	NES	FDR *q*-val
**Mitosis**			
M_PHASE_OF_MITOTIC_CELL_CYCLE	84	-1.689	0.248
MITOSIS	81	-1.653	0.244
CELL_CYCLE_PROCESS	186	-1.621	0.250
REGULATION_OF_MITOSIS	40	-1.569	0.195
**Cellular component organization and assembly**			
ORGANELLE_LOCALIZATION	24	-1.653	0.232
MICROTUBULE_BASED_PROCESS	81	-1.609	0.228
POSITIVE_REGULATION_OF_CELLULAR_COMPONENT_ORGANIZATION_AND_BIOGENESIS	36	-1.620	0.241
ACTIN_CYTOSKELETON_ORGANIZATION_AND_BIOGENESIS	105	-1.595	0.207
ORGANELLE_ORGANIZATION_AND_BIOGENESIS	462	-1.590	0.191
ACTIN_FILAMENT_BASED_PROCESS	115	-1.583	0.186
CELLULAR_COMPONENT_ASSEMBLY	286	-1.549	0.183
**Developmental processes**			
ECTODERM_DEVELOPMENT	80	-1.612	0.233
EPIDERMIS_DEVELOPMENT	71	-1.566	0.187
**Protein modification**			
PROTEIN_FOLDING	57	-1.607	0.224
UBIQUITIN_CYCLE	48	-1.606	0.216
PROTEIN_MODIFICATION_BY_SMALL_PROTEIN_CONJUGATION	43	-1.588	0.186
PROTEIN_UBIQUITINATION	40	-1.556	0.183
**RNA metabolic process**			
MRNA_METABOLIC_PROCESS	66	-1.556	0.191
RIBONUCLEOTIDE_METABOLIC_PROCESS	15	-1.542	0.182
**Chromosome organization**			
CHROMOSOME_ORGANIZATION_AND_BIOGENESIS	118	-1.550	0.186
NUCLEOCYTOPLASMIC_TRANSPORT	85	-1.547	0.181
NUCLEAR_TRANSPORT	86	-1.540	0.181
MACROMOLECULAR_COMPLEX_ASSEMBLY	269	-1.533	0.186
**Others**			
DNA_RECOMBINATION	45	-1.603	0.216
ONE_CARBON_COMPOUND_METABOLIC_PROCESS	26	-1.597	0.213
ACTIVATION_OF_MAPK_ACTIVITY	41	-1.595	0.211
I_KAPPAB_KINASE_NF_KAPPAB_CASCADE	110	-1.594	0.201
AXON_GUIDANCE	22	-1.568	0.191
CELLULAR_BIOSYNTHETIC_PROCESS	317	-1.564	0.189
CELL_PROJECTION_BIOGENESIS	25	-1.556	0.187

C

BIOLOGICAL PROCESS	SIZE	NES	FDR *q*-val
**Response to stimulus**			
CELLULAR_RESPONSE_TO_STIMULUS	19	1.945	0.050
RESPONSE_TO_EXTRACELLULAR_STIMULUS	33	1.771	0.055
RESPONSE_TO_NUTRIENT_LEVELS	29	1.742	0.075
**Lipid metabolic process**			
LIPID_BIOSYNTHETIC_PROCESS	95	1.920	0.050
GLYCEROPHOSPHOLIPID_METABOLIC_PROCESS	46	1.879	0.050
MEMBRANE_LIPID_BIOSYNTHETIC_PROCESS	47	1.869	0.050
CARBOXYLIC_ACID_METABOLIC_PROCESS	175	1.815	0.056
ORGANIC_ACID_METABOLIC_PROCESS	177	1.822	0.057
ALCOHOL_METABOLIC_PROCESS	87	1.811	0.054
LIPID_METABOLIC_PROCESS	318	1.797	0.053
CELLULAR_LIPID_CATABOLIC_PROCESS	35	1.783	0.053
CELLULAR_LIPID_METABOLIC_PROCESS	250	1.775	0.052
LIPID_CATABOLIC_PROCESS	38	1.682	0.092
**Negative regulation of transcription**			
NEGATIVE_REGULATION_OF_TRANSCRIPTION	181	1.815	0.055
NEGATIVE_REGULATION_OF_NUCLEOBASENUCLEOSIDENUCLEOTIDE_AND_NUCLEIC_ACID_METABOLIC_PROCESS	201	1.775	0.052
NEGATIVE_REGULATION_OF_TRANSCRIPTION_DNA_DEPENDENT	128	1.721	0.081
NEGATIVE_REGULATION_OF_RNA_METABOLIC_PROCESS	128	1.721	0.077
NEGATIVE_REGULATION_OF_TRANSCRIPTION_FACTOR_ACTIVITY	15	1.716	0.076
**Catabolic process**			
CELLULAR_CATABOLIC_PROCESS	212	1.811	0.054
CELLULAR_CARBOHYDRATE_CATABOLIC_PROCESS	23	1.694	0.089
CATABOLIC_PROCESS	224	1.688	0.092
**Carbohydrate metabolic process**			
CELLULAR_CARBOHYDRATE_METABOLIC_PROCESS	126	1.803	0.053
GLUCOSE_METABOLIC_PROCESS	28	1.670	0.097
**Negative regulation of metabolic process**			
NEGATIVE_REGULATION_OF_CELLULAR_METABOLIC_PROCESS	249	1.750	0.068
NEGATIVE_REGULATION_OF_METABOLIC_PROCESS	252	1.746	0.071
**Negative regulation of binding**			
NEGATIVE_REGULATION_OF_DNA_BINDING	17	1.730	0.075
NEGATIVE_REGULATION_OF_BINDING	18	1.668	0.097
**Others**			
ICOSANOID_METABOLIC_PROCESS	17	1.804	0.053
HOMEOSTASIS_OF_NUMBER_OF_CELLS	20	1.757	0.062
NEGATIVE_REGULATION_OF_CELL_CYCLE	79	1.696	0.084

D

BIOLOGICAL PROCESS	SIZE	NES	FDR *q*-val
**Cellular response to DNA damage stimulus**			
DNA_RECOMBINATION	45	-1.615	0.243
DNA_DAMAGE_RESPONSESIGNAL_TRANSDUCTION	34	-1.605	0.239
DNA_DAMAGE_CHECKPOINT	20	-1.598	0.247
DNA_REPAIR	122	-1.582	0.241
NUCLEOTIDE_EXCISION_REPAIR	20	-1.575	0.227
**Mitosis**			
CELL_CYCLE_PHASE	166	-1.597	0.237
MITOSIS	81	-1.590	0.239
M_PHASE	110	-1.583	0.247
CELL_CYCLE_PROCESS	186	-1.580	0.234
M_PHASE_OF_MITOTIC_CELL_CYCLE	84	-1.559	0.217
CELL_CYCLE_GO_0007049	306	-1.548	0.218
REGULATION_OF_MITOSIS	40	-1.537	0.218
MITOTIC_CELL_CYCLE	151	-1.528	0.200
**Nucleic acid metabolism**			
AROMATIC_COMPOUND_METABOLIC_PROCESS	27	-1.596	0.232
DNA_METABOLIC_PROCESS	245	-1.573	0.223
COENZYME_METABOLIC_PROCESS	37	-1.556	0.222
TRANSCRIPTION_FROM_RNA_POLYMERASE_III_PROMOTER	19	-1.548	0.216
DNA_REPLICATION	94	-1.531	0.211
REGULATION_OF_GENE_EXPRESSION_EPIGENETIC	29	-1.520	0.197
**Ribonucleoprotein complex biogenesis**			
RIBONUCLEOPROTEIN_COMPLEX_BIOGENESIS_AND_ASSEMBLY	76	-1.565	0.218
MRNA_METABOLIC_PROCESS	66	-1.530	0.207
REGULATION_OF_TRANSLATIONAL_INITIATION	31	-1.530	0.203
TRANSLATION	178	-1.549	0.221
REGULATION_OF_PROTEIN_METABOLIC_PROCESS	173	-1.513	0.205
**Apoptosis**			
NEGATIVE_REGULATION_OF_APOPTOSIS	149	-1.554	0.217
INDUCTION_OF_APOPTOSIS_BY_EXTRACELLULAR_SIGNALS	27	-1.508	0.210
**Others**			
MICROTUBULE_CYTOSKELETON_ORGANIZATION_AND_BIOGENESIS	34	-1.623	0.248
NEGATIVE_REGULATION_OF_PROGRAMMED_CELL_DEATH	150	-1.571	0.218
REGULATION_OF_ANATOMICAL_STRUCTURE_MORPHOGENESIS	25	-1.543	0.216
CELL_PROJECTION_BIOGENESIS	25	-1.525	0.197

E

MOLECULAR FUNCTION	SIZE	NES	FDR *q*-val
**Oxidoreductase activity**			
GO_OXIDOREDUCTASE_ACTIVITY_ACTING_ON_PAIRED_DONORS_WITH_INCORPORATION_OR_REDUCTION_OF_MOLECULAR_OXYGEN_NAD_P_H_AS_ONE_DONOR_AND_INCORPORATION_OF_ONE_ATOM_OF_OXYGEN	36	2.062	0.039
GO_OXIDOREDUCTASE_ACTIVITY_ACTING_ON_THE_ALDEHYDE_OR_OXO_GROUP_OF_DONORS_NAD_OR_NADP_AS_ACCEPTOR	36	2.023	0.039
GO_OXIDOREDUCTASE_ACTIVITY_ACTING_ON_NAD_P_H_QUINONE_OR_SIMILAR_COMPOUND_AS_ACCEPTOR	51	1.903	0.039
GO_OXIDOREDUCTASE_ACTIVITY_ACTING_ON_THE_ALDEHYDE_OR_OXO_GROUP_OF_DONORS	46	1.888	0.052
GO_OXIDOREDUCTASE_ACTIVITY_ACTING_ON_NAD_P_H	91	1.887	0.051
GO_OXIDOREDUCTASE_ACTIVITY_ACTING_ON_A_SULFUR_GROUP_OF_DONORS	48	1.799	0.063
GO_OXIDOREDUCTASE_ACTIVITY_ACTING_ON_CH_OH_GROUP_OF_DONORS	131	1.840	0.054
GO_OXIDOREDUCTASE_ACTIVITY_ACTING_ON_THE_CH_CH_GROUP_OF_DONORS	56	1.765	0.063
GO_OXIDOREDUCTASE_ACTIVITY_ACTING_ON_THE_CH_OH_GROUP_OF_DONORS_NAD_OR_NADP_AS_ACCEPTOR	110	1.759	0.060
GO_OXIDOREDUCTASE_ACTIVITY_ACTING_ON_PAIRED_DONORS_WITH_INCORPORATION_OR_REDUCTION_OF_MOLECULAR_OXYGEN	143	1.726	0.062
GO_INTRAMOLECULAR_OXIDOREDUCTASE_ACTIVITY	51	1.733	0.064
GO_INTRAMOLECULAR_OXIDOREDUCTASE_ACTIVITY_TRANSPOSING_S_S_BONDS	22	1.727	0.063
**Binding activity**			
GO_COFACTOR_BINDING	255	1.987	0.039
GO_COENZYME_BINDING	172	1.888	0.047
GO_NADP_BINDING	42	1.851	0.055
GO_GDP_BINDING	51	1.843	0.055
GO_FLAVIN_ADENINE_DINUCLEOTIDE_BINDING	72	1.789	0.060
GO_GABA_RECEPTOR_BINDING	15	1.760	0.062
GO_APOLIPOPROTEIN_BINDING	15	1.759	0.060
GO_GTP_DEPENDENT_PROTEIN_BINDING	17	1.715	0.066
**Lyase activity**			
GO_CARBON_CARBON_LYASE_ACTIVITY	49	2.012	0.039
GO_CARBOXY_LYASE_ACTIVITY	35	1.848	0.057
**Transferase activity**			
GO_GLUTATHIONE_TRANSFERASE_ACTIVITY	34	1.851	0.053
GO_TRANSFERASE_ACTIVITY_TRANSFERRING_ALKYL_OR_ARYL_OTHER_THAN_METHYL_GROUPS	62	1.756	0.059
**Transporter activity**			
GO_MAGNESIUM_ION_TRANSMEMBRANE_TRANSPORTER_ACTIVITY	15	1.791	0.062
GO_COFACTOR_TRANSPORTER_ACTIVITY	21	1.708	0.068
**Others**			
GO_LIPOPROTEIN_PARTICLE_RECEPTOR_ACTIVITY	15	1.903	0.039
GO_ALDO_KETO_REDUCTASE_NADP_ACTIVITY	26	1.784	0.059
GO_ELECTRON_CARRIER_ACTIVITY	108	1.783	0.058
GO_MONOOXYGENASE_ACTIVITY	91	1.721	0.065

F

MOLECULAR FUNCTION	SIZE	NES	FDR *q*-val
**Binding activity**			
GO_PHOSPHOLIPASE_BINDING	18	-1.960	0.154
GO_CHAPERONE_BINDING	77	-1.829	0.207
GO_METAL_CLUSTER_BINDING	62	-1.796	0.182
GO_HEAT_SHOCK_PROTEIN_BINDING	84	-1.765	0.219
GO_MRNA_BINDING	139	-1.740	0.242
GO_FIBROBLAST_GROWTH_FACTOR_BINDING	23	-1.722	0.248
GO_R_SMAD_BINDING	21	-1.700	0.242
GO_PROTEIN_BINDING_INVOLVED_IN_CELL_ADHESION	17	-1.698	0.218
GO_SH3_DOMAIN_BINDING	115	-1.653	0.174
GO_4_IRON_4_SULFUR_CLUSTER_BINDING	41	-1.653	0.168
GO_DNA_BINDING_BENDING	20	-1.637	0.173
GO_RIBOSOME_BINDING	41	-1.620	0.177
GO_MITOGEN_ACTIVATED_PROTEIN_KINASE_BINDING	24	-1.610	0.181
**ATPase activity**			
GO_ATPASE_ACTIVATOR_ACTIVITY	17	-1.915	0.192
GO_ATPASE_REGULATOR_ACTIVITY	30	-1.887	0.141
**Transporter activity**			
GO_SODIUM_INDEPENDENT_ORGANIC_ANION_TRANSMEMBRANE_TRANSPORTER_ACTIVITY	21	-1.801	0.211
GO_PROTEIN_TRANSPORTER_ACTIVITY	96	-1.641	0.173
**Transferase activity**			
GO_PROTEIN_METHYLTRANSFERASE_ACTIVITY	74	-1.696	0.211
GO_RNA_METHYLTRANSFERASE_ACTIVITY	35	-1.696	0.199
GO_S_ADENOSYLMETHIONINE_DEPENDENT_METHYLTRANSFERASE_ACTIVITY	120	-1.688	0.194
GO_TRNA_METHYLTRANSFERASE_ACTIVITY	17	-1.683	0.193
GO_N_METHYLTRANSFERASE_ACTIVITY	73	-1.662	0.192
GO_LYSINE_N_METHYLTRANSFERASE_ACTIVITY	50	-1.656	0.181
**Others**			
GO_LIGASE_ACTIVITY_FORMING_CARBON_OXYGEN_BONDS	44	-1.698	0.229
GO_NUCLEOSIDE_TRIPHOSPHATASE_REGULATOR_ACTIVITY	307	-1.692	0.192
GO_NUCLEOBASE_CONTAINING_COMPOUND_KINASE_ACTIVITY	44	-1.666	0.198
GO_CYCLIN_DEPENDENT_PROTEIN_KINASE_ACTIVITY	21	-1.662	0.185
GO_RHO_GUANYL_NUCLEOTIDE_EXCHANGE_FACTOR_ACTIVITY	70	-1.649	0.167
GO_EXONUCLEASE_ACTIVITY	76	-1.635	0.168
GO_GALACTOSYLTRANSFERASE_ACTIVITY	32	-1.610	0.186

G

MOLECULAR FUNCTION	SIZE	NES	FDR *q*-val
**Binding activity**			
GO_RNA_POLYMERASE_II_ACTIVATING_TRANSCRIPTION_FACTOR_BINDING	34	1.959	0.054
GO_PEPTIDE_ANTIGEN_BINDING	29	1.894	0.054
GO_HMG_BOX_DOMAIN_BINDING	18	1.865	0.054
GO_NADP_BINDING	42	1.846	0.086
GO_ACTIVATING_TRANSCRIPTION_FACTOR_BINDING	55	1.826	0.077
GO_TRANSCRIPTIONAL_REPRESSOR_ACTIVITY_RNA_POLYMERASE_II_ACTIVATING_TRANSCRIPTION_FACTOR_BINDING	53	1.805	0.108
GO_SYNTAXIN_BINDING	90	1.782	0.112
GO_COFACTOR_BINDING	255	1.748	0.128
GO_SNARE_BINDING	123	1.732	0.122
GO_FLAVIN_ADENINE_DINUCLEOTIDE_BINDING	72	1.719	0.147
GO_POLYUBIQUITIN_BINDING	37	1.712	0.146
GO_COENZYME_BINDING	172	1.711	0.140
GO_GDP_BINDING	51	1.708	0.140
GO_STEROID_BINDING	89	1.686	0.130
GO_UBIQUITIN_LIKE_PROTEIN_BINDING	112	1.651	0.136
**Endopeptidase activity**			
GO_CALCIUM_DEPENDENT_CYSTEINE_TYPE_ENDOPEPTIDASE_ACTIVITY	20	1.977	0.054
GO_CYSTEINE_TYPE_ENDOPEPTIDASE_ACTIVITY	85	1.667	0.125
**Lipase activity**			
GO_TRIGLYCERIDE_LIPASE_ACTIVITY	20	1.799	0.102
GO_LIPASE_ACTIVITY	114	1.664	0.122
**Transferase activity**			
GO_GLUTATHIONE_TRANSFERASE_ACTIVITY	34	1.764	0.134
GO_NAD_ADP_RIBOSYLTRANSFERASE_ACTIVITY	26	1.670	0.127
**Oxidoreductase activity**			
GO_OXIDOREDUCTASE_ACTIVITY_ACTING_ON_PAIRED_DONORS_WITH_INCORPORATION_OR_REDUCTION_OF_MOLECULAR_OXYGEN	143	1.717	0.142
GO_OXIDOREDUCTASE_ACTIVITY_ACTING_ON_THE_CH_CH_GROUP_OF_DONORS_NAD_OR_NADP_AS_ACCEPTOR	24	1.703	0.139
GO_OXIDOREDUCTASE_ACTIVITY_ACTING_ON_THE_CH_CH_GROUP_OF_DONORS	56	1.703	0.135
GO_OXIDOREDUCTASE_ACTIVITY_ACTING_ON_CH_OH_GROUP_OF_DONORS	131	1.692	0.138
GO_OXIDOREDUCTASE_ACTIVITY_ACTING_ON_A_HEME_GROUP_OF_DONORS	24	1.692	0.134
**Others**			
GO_PROTEIN_SERINE_THREONINE_KINASE_INHIBITOR_ACTIVITY	30	1.835	0.080
GO_DEMETHYLASE_ACTIVITY	24	1.726	0.135
GO_PROTEIN_SELF_ASSOCIATION	44	1.692	0.131
GO_LYSOPHOSPHOLIPASE_ACTIVITY	19	1.687	0.131
GO_ALDEHYDE_DEHYDROGENASE_NAD_ACTIVITY	19	1.674	0.130

H

MOLECULAR FUNCTION	SIZE	NES	FDR *q*-val
**Binding activity**			
GO_DAMAGED_DNA_BINDING	63	-1.821	0.210
GO_RRNA_BINDING	56	-1.729	0.225
GO_GTP_RHO_BINDING	15	-1.710	0.203
GO_FK506_BINDING	18	-1.655	0.194
GO_MACROLIDE_BINDING	18	-1.655	0.211
GO_SINGLE_STRANDED_DNA_BINDING	78	-1.648	0.174
GO_MRNA_BINDING	139	-1.630	0.188
GO_PHOSPHOLIPASE_BINDING	18	-1.580	0.182
GO_DOUBLE_STRANDED_RNA_BINDING	62	-1.551	0.200
**Transferase activity**			
GO_NUCLEOTIDYLTRANSFERASE_ACTIVITY	119	-1.814	0.178
GO_RNA_METHYLTRANSFERASE_ACTIVITY	35	-1.675	0.207
GO_PHOSPHOTRANSFERASE_ACTIVITY_PHOSPHATE_GROUP_AS_ACCEPTOR	34	-1.648	0.183
GO_TRANSFERASE_ACTIVITY_TRANSFERRING_ONE_CARBON_GROUPS	188	-1.628	0.181
GO_PROTEIN_METHYLTRANSFERASE_ACTIVITY	74	-1.559	0.202
GO_S_ADENOSYLMETHIONINE_DEPENDENT_METHYLTRANSFERASE_ACTIVITY	120	-1.555	0.200
**Polymerase activity**			
GO_DNA_DIRECTED_DNA_POLYMERASE_ACTIVITY	26	-1.695	0.192
GO_DNA_POLYMERASE_ACTIVITY	34	-1.660	0.219
GO_RNA_POLYMERASE_ACTIVITY	43	-1.590	0.202
**Helicase activity**			
GO_HELICASE_ACTIVITY	147	-1.639	0.184
GO_RNA_HELICASE_ACTIVITY	65	-1.581	0.186
GO_PURINE_NTP_DEPENDENT_HELICASE_ACTIVITY	94	-1.560	0.206
**Others**			
GO_DNA_N_GLYCOSYLASE_ACTIVITY	15	-1.859	0.141
GO_NUCLEOBASE_CONTAINING_COMPOUND_KINASE_ACTIVITY	44	-1.710	0.228
GO_BASAL_TRANSCRIPTION_MACHINERY_BINDING	26	-1.615	0.197
GO_NUCLEASE_ACTIVITY	190	-1.601	0.200
GO_LIGASE_ACTIVITY_FORMING_CARBON_NITROGEN_BONDS	53	-1.587	0.198
GO_STRUCTURAL_CONSTITUENT_OF_RIBOSOME	205	-1.583	0.194
GO_RECEPTOR_SIGNALING_COMPLEX_SCAFFOLD_ACTIVITY	23	-1.582	0.190
GO_TRANSLATION_INITIATION_FACTOR_BINDING	27	-1.549	0.198
GO_ATPASE_ACTIVITY_COUPLED	307	-1.541	0.203

I

CELLULAR COMPONENT	SIZE	NES	FDR *q*-val
**Microbody**			
GO_MICROBODY	130	1.884	0.157
GO_MICROBODY_PART	90	1.641	0.117
GO_MICROBODY_MEMBRANE	56	1.638	0.118
**Endosomal-lysosomal system**			
GO_AUTOPHAGOSOME	74	1.867	0.148
GO_PRE_AUTOPHAGOSOMAL_STRUCTURE	24	1.783	0.166
GO_ENDOSOMAL_PART	409	1.780	0.143
GO_VACUOLAR_LUMEN	113	1.697	0.155
GO_LYSOSOMAL_LUMEN	86	1.671	0.118
GO_LATE_ENDOSOME_MEMBRANE	98	1.659	0.123
GO_LYTIC_VACUOLE_MEMBRANE	258	1.658	0.121
GO_PRE_AUTOPHAGOSOMAL_STRUCTURE_MEMBRANE	15	1.649	0.118
GO_LATE_ENDOSOME	204	1.637	0.117
**Motile cilium**			
GO_CILIARY_TRANSITION_ZONE	21	1.727	0.165
GO_MOTILE_CILIUM	98	1.677	0.121
GO_PHOTORECEPTOR_CONNECTING_CILIUM	30	1.673	0.119
GO_CILIARY_TIP	39	1.624	0.122
GO_DYNEIN_COMPLEX	42	1.572	0.142
GO_SPERM_FLAGELLUM	53	1.595	0.135
**Intrinsic component of organelle membrane**			
GO_INTRINSIC_COMPONENT_OF_ENDOPLASMIC_RETICULUM_MEMBRANE	131	1.708	0.162
GO_INTRINSIC_COMPONENT_OF_MITOCHONDRIAL_OUTER_MEMBRANE	21	1.695	0.145
GO_INTRINSIC_COMPONENT_OF_ORGANELLE_MEMBRANE	261	1.689	0.136
**Others**			
GO_PIGMENT_GRANULE	102	1.820	0.171
GO_LIPID_PARTICLE	57	1.716	0.165
GO_GLIAL_CELL_PROJECTION	15	1.684	0.129
GO_PRESYNAPTIC_ACTIVE_ZONE	29	1.682	0.124
GO_PROTEIN_DNA_COMPLEX	166	1.656	0.118
GO_CUL3_RING_UBIQUITIN_LIGASE_COMPLEX	61	1.654	0.116
GO_CIS_GOLGI_NETWORK	40	1.609	0.131
GO_BLOC_COMPLEX	19	1.583	0.137
GO_OXIDOREDUCTASE_COMPLEX	91	1.577	0.140

J

CELLULAR COMPONENT	SIZE	NES	FDR *q*-val
**Spindle pole**			
GO_CHROMOSOME_CENTROMERIC_REGION	162	-1.638	0.235
GO_SPINDLE_MIDZONE	27	-1.627	0.249
GO_CONDENSED_CHROMOSOME_CENTROMERIC_REGION	93	-1.624	0.240
GO_KINETOCHORE	110	-1.586	0.239
GO_KINESIN_COMPLEX	55	-1.577	0.220
GO_SPINDLE_POLE	111	-1.577	0.215
GO_MICROTUBULE_ORGANIZING_CENTER_PART	128	-1.563	0.212
GO_CHROMOSOME_TELOMERIC_REGION	146	-1.544	0.195
GO_NUCLEAR_CHROMOSOME_TELOMERIC_REGION	117	-1.540	0.190
GO_CONDENSED_NUCLEAR_CHROMOSOME_CENTROMERIC_REGION	17	-1.532	0.184
**Nucleolar part**			
GO_NUCLEOLAR_PART	61	-1.595	0.248
GO_NUCLEAR_MATRIX	93	-1.590	0.242
GO_TRANSCRIPTIONAL_REPRESSOR_COMPLEX	73	-1.560	0.203
**Ribosome**			
GO_SMALL_NUCLEAR_RIBONUCLEOPROTEIN_COMPLEX	57	-1.586	0.233
GO_CYTOSOLIC_RIBOSOME	106	-1.573	0.209
GO_LARGE_RIBOSOMAL_SUBUNIT	91	-1.546	0.199
GO_RNA_POLYMERASE_COMPLEX	118	-1.543	0.191
GO_RIBOSOME	215	-1.531	0.177
**Ruffle**			
GO_RUFFLE	155	-1.579	0.225
GO_RUFFLE_MEMBRANE	80	-1.549	0.210
**Cell junction**			
GO_ANCHORING_JUNCTION	478	-1.547	0.204
GO_CELL_SUBSTRATE_JUNCTION	390	-1.536	0.183
GO_GAP_JUNCTION	29	-1.532	0.180
**Others**			
GO_CHD_TYPE_COMPLEX	17	-1.640	0.243
GO_PRONUCLEUS	15	-1.605	0.250
GO_TRANSFERASE_COMPLEX_TRANSFERRING_PHOSPHORUS_CONTAINING_GROUPS	225	-1.581	0.230
GO_PLATELET_ALPHA_GRANULE_LUMEN	55	-1.547	0.207
GO_RETROMER_COMPLEX	21	-1.539	0.187
GO_MITOCHONDRIAL_MATRIX	394	-1.530	0.176
GO_DNA_DIRECTED_RNA_POLYMERASE_II_HOLOENZYME	93	-1.530	0.173

K

CELLULAR COMPONENT	SIZE	NES	FDR *q*-val
**ER to Golgi transport**			
GO_ER_TO_GOLGI_TRANSPORT_VESICLE	70	1.946	0.076
GO_AP_TYPE_MEMBRANE_COAT_ADAPTOR_COMPLEX	37	1.858	0.079
GO_COATED_VESICLE_MEMBRANE	135	1.821	0.060
GO_TRANSPORT_VESICLE	326	1.799	0.055
GO_ER_TO_GOLGI_TRANSPORT_VESICLE_MEMBRANE	54	1.789	0.052
GO_TRANS_GOLGI_NETWORK	187	1.780	0.052
GO_SNARE_COMPLEX	53	1.734	0.062
GO_COPI_COATED_VESICLE	22	1.733	0.061
GO_TRANSPORT_VESICLE_MEMBRANE	148	1.729	0.061
GO_COATED_VESICLE	230	1.706	0.063
**Endosomal-lysosomal system**			
GO_AUTOPHAGOSOME	74	1.932	0.056
GO_PRE_AUTOPHAGOSOMAL_STRUCTURE	24	1.888	0.069
GO_LATE_ENDOSOME_MEMBRANE	98	1.853	0.072
GO_BLOC_COMPLEX	19	1.826	0.063
GO_LATE_ENDOSOME	204	1.791	0.053
GO_ENDOSOMAL_PART	409	1.784	0.051
GO_AUTOPHAGOSOME_MEMBRANE	25	1.781	0.053
GO_EARLY_ENDOSOME_MEMBRANE	107	1.737	0.058
GO_EARLY_ENDOSOME	288	1.731	0.060
GO_LYTIC_VACUOLE_MEMBRANE	258	1.701	0.066
**Intrinsic component of organelle membrane**			
GO_INTRINSIC_COMPONENT_OF_ENDOPLASMIC_RETICULUM_MEMBRANE	131	1.806	0.058
GO_INTRINSIC_COMPONENT_OF_ORGANELLE_MEMBRANE	261	1.742	0.055
GO_INTRINSIC_COMPONENT_OF_MITOCHONDRIAL_OUTER_MEMBRANE	21	1.690	0.068
**Motile cilium**			
GO_CILIARY_TIP	39	1.799	0.056
GO_PHOTORECEPTOR_CONNECTING_CILIUM	30	1.761	0.053
GO_DYNEIN_COMPLEX	42	1.760	0.052
**Others**			
GO_PHOSPHATASE_COMPLEX	46	1.865	0.061
GO_MICROBODY	130	1.828	0.067
GO_LIPID_PARTICLE	57	1.741	0.055
GO_ORGANELLE_SUBCOMPARTMENT	301	1.700	0.066

L

CELLULAR COMPONENT	SIZE	NES	FDR *q*-val
**Ribosome**			
GO_CYTOSOLIC_RIBOSOME	106	-1.996	0.070
GO_CYTOSOLIC_LARGE_RIBOSOMAL_SUBUNIT	58	-1.893	0.106
GO_REPLISOME	28	-1.873	0.083
GO_LARGE_RIBOSOMAL_SUBUNIT	91	-1.633	0.237
GO_SMALL_RIBOSOMAL_SUBUNIT	66	-1.613	0.182
GO_RIBOSOMAL_SUBUNIT	157	-1.609	0.168
GO_RIBOSOME	215	-1.569	0.145
GO_CYTOPLASMIC_EXOSOME_RNASE_COMPLEX	15	-1.568	0.143
GO_POLYSOME	41	-1.586	0.151
**Mitochondrion**			
GO_MITOCHONDRIAL_MATRIX	394	-1.625	0.224
GO_MITOCHONDRIAL_PROTEIN_COMPLEX	125	-1.576	0.143
GO_INTRINSIC_COMPONENT_OF_MITOCHONDRIAL_INNER_MEMBRANE	17	-1.586	0.146
GO_INNER_MITOCHONDRIAL_MEMBRANE_PROTEIN_COMPLEX	95	-1.555	0.151
**Nuclear part**			
GO_NUCLEAR_PERIPHERY	115	-1.615	0.191
GO_RNA_POLYMERASE_COMPLEX	118	-1.611	0.175
GO_DNA_DIRECTED_RNA_POLYMERASE_II_HOLOENZYME	93	-1.600	0.161
GO_REPLICATION_FORK	57	-1.576	0.141
**Spindle pole**			
GO_CHROMOSOMAL_REGION	303	-1.588	0.157
GO_CHROMOSOME_CENTROMERIC_REGION	162	-1.586	0.155
GO_KINETOCHORE	110	-1.579	0.144
GO_CONDENSED_NUCLEAR_CHROMOSOME	79	-1.551	0.148
GO_SPINDLE	259	-1.544	0.153
**Others**			
GO_CARBOXY_TERMINAL_DOMAIN_PROTEIN_KINASE_COMPLEX	22	-1.824	0.098
GO_CUL4_RING_E3_UBIQUITIN_LIGASE_COMPLEX	18	-1.733	0.211
GO_ORGANELLE_ENVELOPE_LUMEN	78	-1.623	0.213
GO_ORGANELLE_INNER_MEMBRANE	490	-1.622	0.200
GO_CYCLIN_DEPENDENT_PROTEIN_KINASE_HOLOENZYME_COMPLEX	27	-1.596	0.159
GO_CYTOSOLIC_PART	212	-1.591	0.161
GO_HETEROTRIMERIC_G_PROTEIN_COMPLEX	32	-1.579	0.148
GO_METHYLTRANSFERASE_COMPLEX	85	-1.573	0.142
