# Supplementary material for: Molecular action of isoflavone genistein in the human epithelial cell line HaCaT
Source: PLoS One. 2018 Feb 14;13(2):e0192297. doi: 10.1371/journal.pone.0192297 (PMC5812592; doi:10.1371/journal.pone.0192297)
Supplement: S1 Table — Keratinocytes were treated with different concentrations of genistein (GEN) for 24 hours, 48 hours (cytotoxicity assay), and 7 days (proliferation assay); afterward the percentage of cell survival was determined. Results are expressed as mean values of three experiments with error bars indicating standard deviation. (DOCX) [file pone.0192297.s005.docx]

| Conditions | Exposure time | | |
| --- | --- | --- | --- |
|  | 24 h | 48 h | 7 d |
| 0.05% DMSO | 0.887 ± 0.042 | 0.908 ± 0.150 | 1.047 ± 0.056 |
| 10 µM GEN | 0.870 ± 0.034 | 0.851 ± 0.004 | 0.946 ± 0.120 |
| 30 µM GEN | 0.882 ± 0.008 | 0.767 ± 0.095 | 0.219 ± 0.001 |
| 60 µM GEN | 0.835 ± 0.046 | 0.604 ± 0.040 | 0.235 ± 0.019 |
| 100 µM GEN | 0.847 ± 0.066 | 0.590 ± 0.083 | 0.214 ± 0.014 |
