# Supplementary material for: Molecular action of isoflavone genistein in the human epithelial cell line HaCaT
Source: PLoS One. 2018 Feb 14;13(2):e0192297. doi: 10.1371/journal.pone.0192297 (PMC5812592; doi:10.1371/journal.pone.0192297)
Supplement: S2 Table — Cytotoxicity is expressed as LC25, 50 or 75 (i.e., concentration of the tested drug [μM]) that is lethal to 25%, 50%, or 75% of HaCaT cells, respectively, in a culture exposed to the drug for 24 and 48 hours. Antiproliferative activity is expressed as IC25, 50 or 75 (i.e., concentration of the tested drug [μM]) that causes 25%, 50%, or 75% inhibition of keratinocyte proliferation, respectively, in a culture exposed to the drug for 7 days. (DOCX) [file pone.0192297.s006.docx]

| Time of exposure | LC25 [µM] | LC50 [µM] | LC75 [µM] |
| --- | --- | --- | --- |
| 24 h | >100 | >100 | >100 |
| 48 h | >100 | >100 | 59 |
|  | IC25 [µM] | IC50 [µM] | IC75 [µM] |
| 7 d | 30 | 21 | 12 |
