# Supplementary material for: Molecular action of isoflavone genistein in the human epithelial cell line HaCaT
Source: PLoS One. 2018 Feb 14;13(2):e0192297. doi: 10.1371/journal.pone.0192297 (PMC5812592; doi:10.1371/journal.pone.0192297)
Supplement: S3 Table — Abbreviations: n: number of samples; GM [CP]: the geometric mean of CP; AM [CP]: the arithmetic mean of CP; Min [CP] and Max [CP]: the extreme values of CP; SD [± CP]: the standard deviation of the CP; CV [% CP]: the coefficient of variance expressed as a percentage on the CP level; Min [x-fold] and Max [x-fold]: the extreme values of expression levels expressed as an absolute x-fold over- or under-regulation coefficient; SD [± x-fold]: standard deviation of the absolute regulation coefficients; coeff. of corr. [r]: coefficient of correlation; coeff. of det. [r2]: coefficient of determination. (DOCX) [file pone.0192297.s007.docx]

| Factor | *18S* | *ACTB* | *ALAS* | *B2M* | *G6PDH* | *GAPDH* | *PGK1* | *RPLP0* | *TBP* | *YWHAZ* | BestKeeper  n = 10 |
| --- | --- | --- | --- | --- | --- | --- | --- | --- | --- | --- | --- |
| n | 8 | 8 | 8 | 8 | 8 | 8 | 8 | 8 | 8 | 8 | 8 |
| GM [CP] | 11.47 | 18.97 | 26.26 | 20.87 | 25.05 | 30.78 | 23.29 | 19.91 | 27.91 | 21.75 | 21.94 |
| AM [CP] | 11.47 | 18.98 | 26.26 | 20.87 | 25.08 | 30.78 | 23.29 | 19.92 | 27.91 | 21.75 | 21.94 |
| Min [CP] | 10.84 | 18.16 | 25.76 | 20.19 | 23.22 | 29.66 | 22.49 | 19.26 | 27.33 | 20.80 | 21.34 |
| Max [CP] | 11.86 | 19.65 | 26.64 | 21.54 | 26.65 | 31.28 | 23.80 | 20.54 | 28.33 | 22.15 | 22.44 |
| SD [±CP] | 0.35 | 0.39 | 0.28 | 0.38 | 0.93 | 0.41 | 0.39 | 0.32 | 0.27 | 0.37 | 0.29 |
| CV [%CP] | 3.01 | 2.04 | 1.08 | 1.82 | 3.72 | 1.32 | 1.68 | 1.62 | 0.97 | 1.71 | 1.34 |
|  | | | | | | | | | | | |
| Min [x-fold] | -1.54 | -1.76 | -1.41 | -1.60 | -3.57 | -2.17 | -1.74 | -1.57 | -1.49 | -1.93 | 1.51 |
| Max [x-fold] | 1.31 | 1.60 | 1.30 | 1.60 | 3.02 | 1.41 | 1.43 | 1.55 | 1.34 | 1.32 | 1.41 |
| SD [± x-fold] | 1.27 | 1.31 | 1.22 | 1.30 | 1.91 | 1.33 | 1.31 | 1.25 | 1.21 | 1.29 | 1.23 |
| Coeff. of corr. [r] | 0.495 | 0.733 | 0.922 | 0.703 | 0.373 | 0.889 | 0.840 | 0.942 | 0.990 | 0.940 | n.a. |
| Coeff. of det. [r^2^] | 0.245 | 0.537 | 0.850 | 0.494 | 0.139 | 0.790 | 0.706 | 0.887 | 0.980 | 0.884 | n.a. |
| *p-value* | 0.211 | 0.039 | 0.001 | 0.052 | 0.365 | 0.003 | 0.009 | 0.001 | 0.001 | 0.001 | n.a. |

Abbreviations: n: number of samples; n.a.: not applicable; GM [CP]: the geometric mean of CP; AM [CP]: the arithmetic mean of CP; Min [CP] and Max [CP]: the extreme values of CP; SD [± CP]: the standard deviation of the CP; CV [% CP]: the coefficient of variance expressed as a percentage on the CP level; Min [x-fold] and Max [x-fold]: the extreme values of expression levels expressed as an absolute x-fold over- or under-regulation coefficient; SD [± x-fold]: standard deviation of the absolute regulation coefficients; coeff. of corr. [r]: coefficient of correlation; coeff. of det. [r^2^]: coefficient of determination.
