# Supplementary material for: Molecular action of isoflavone genistein in the human epithelial cell line HaCaT
Source: PLoS One. 2018 Feb 14;13(2):e0192297. doi: 10.1371/journal.pone.0192297 (PMC5812592; doi:10.1371/journal.pone.0192297)
Supplement: S4 Table — The analysis was made via real-time qRT-PCR in response to various tested “psoriasis-like” activation processes: treatment with the proinflammatory “cytokine mix” (2 ng/mL of IL-1A, IL-17A, IL-22, OSM and TNF-α), co-culture of HaCaT and THP-1, with or without addition of 1 μg/mL lipopolysaccharide (LPS) for 24 hours. mRNA expression levels for the four marker genes were normalized using TBP housekeeping reference and expressed as the fold change for stimulated vs. unstimulated cells. (DOCX) [file pone.0192297.s008.docx]

| Gene symbol | “cytokine mix” | THP-1 | THP-1 + LPS |
| --- | --- | --- | --- |
| *LOR* | 0.5 ± 0.1 | 0.6 ± 0.1 | 0.1 ± 0.1 |
| *KRT10* | 0.2 ± 0.1 | 0.4 ± 0.1 | 0.2 ± 0.1 |
| *S100A7* | 54.2 ± 2.0 | 0.4 ± 0.1 | 0.8 ± 0.1 |
| *S100A9* | 153.6 ± 18.2 | 0.8 ± 0.1 | 2.0 ± 0.4 |
