# Supplementary material for: Molecular action of isoflavone genistein in the human epithelial cell line HaCaT
Source: PLoS One. 2018 Feb 14;13(2):e0192297. doi: 10.1371/journal.pone.0192297 (PMC5812592; doi:10.1371/journal.pone.0192297)
Supplement: S5 Table — Keratinocytes were treated with 0.05% DMSO only (NACT), stimulated with a combination of proinflammatory “cytokine mix” (ACT), stimulated with a combination of proinflammatory “cytokine mix” and treated with 100 μM genistein (GEN), or stimulated with a combination of proinflammatory “cytokine mix” and treated with wortmannin (a PI3K inhibitor) (WORT). (DOCX) [file pone.0192297.s009.docx]

| Activity of PI3 kinase | | | | |
| --- | --- | --- | --- | --- |
| Status | NACT | ACT | GEN | WORT |
| Non-expressing | 4.33 ± 1.82 | 3.58 ± 1.48 | 2.52 ± 0.23 | 18.12 ± 2.97 |
| Activated | 15.10 ± 1.74 | 20.64 ± 6.24 | 16.62 ± 0.78 | 7.52 ± 0.57 |
| Inactivated | 80.55 ± 0.65 | 75.36 ± 6.84 | 80.87 ± 0.81 | 74.32 ± 3.18 |
| Activity of *PI3K* gene | | | | |
| Relative mRNA level of *PI3K* | n.d. | 1.96 ± 0.16 | 1.29 ± 0.1 | n.d. |
