# Supplementary material for: Molecular action of isoflavone genistein in the human epithelial cell line HaCaT
Source: PLoS One. 2018 Feb 14;13(2):e0192297. doi: 10.1371/journal.pone.0192297 (PMC5812592; doi:10.1371/journal.pone.0192297)
Supplement: S6 Table — (B) Analysis of ROS were additionally performed by fluorescent cell analyzer. The data are presented as the means ± standard deviation (SD) from three independent experiments. Significant differences (p ≤ 0.05) between cell populations not expressing intracellular ROS (ROS [–]) and expressing intracellular ROS (ROS [+]) were observed for all tested conditions, except for LPS where cell numbers of ROS (-) and ROS (+) were comparable. Statistical analysis was performed using ANOVA with Tukey’s HSD test. (DOCX) [file pone.0192297.s010.docx]

| Conditions | ROS (-) Cells | ROS (+) Cells |
| --- | --- | --- |
| NACT | 73.83  72.2  73.9 | 25.7  26.93  25.63 |
| ACT 2ng/ml | 71.83  68.47  71.6 | 27.57  30.8  28.2 |
| ACT 5ng/ml | 68.73  73.3  75.2 | 30.83  26.2  24.43 |
| ACT2 + GEN | 68  64.4  65.27 | 31.37  34.87  34.03 |
| GEN | 68.77  70.2  70.6 | 30.87  29.57  28.57 |
| ACT + NAC | 74.78  79.03  72.7 | 22.96  19.17  25.88 |
| TNF-α | 39.57  40.13  44.47 | 60.2  59.17  55.07 |
| TNF-α + GEN | 59.37  65.03  63.8 | 40.37  34.53  35.4 |
| LPS | 52.93  51.6  49.03 | 46.73  47.83  50.33 |
| LPS + GEN | 72.03  65.8  64.7 | 27.3  33.53  34.6 |
