# Supplementary material for: Molecular action of isoflavone genistein in the human epithelial cell line HaCaT
Source: PLoS One. 2018 Feb 14;13(2):e0192297. doi: 10.1371/journal.pone.0192297 (PMC5812592; doi:10.1371/journal.pone.0192297)
Supplement: S7 Table — DMSO-treated, unstimulated cells were used as control (NACT). The data are presented as the means ± standard deviation (SD) from three independent experiments. Comparisons among groups were performed using a one-way ANOVA with Tukey’s HSD test. (DOCX) [file pone.0192297.s011.docx]

| Conditions | Absorbance 450 nm | | |
| --- | --- | --- | --- |
|  | IL8 | IL20 | CCL2 |
| NACT | 0.163  0.162  0.160  0.160  0.147  0.122 | 0.143  0.139  0.136  0.138  0.136  0.153 | 0.248  0.269  0.260  0.203  0.201  0.234 |
| GEN | 0.073  0.071  0.082  0.072  0.143  0.095 | 0.142  0.136  0.132  0.135  0.137  0.14 | 0.139  0.147  0.140  0.158  0.229  0.159 |
| MTX | 0.147  0.106  0.143  0.122  0.140  0.137 | 0.139  0.134  0.134  0.14  0.175  0.135 | 0.209  0.267  0.255  0.218  0.194  0.212 |
| ACT | 1.425  1.368  0.993  1.228  1.082  1.332 | 0.151  0.153  0.176  0.186  0.171  0.151 | 2.474  2.313  2.243  2.327  2.367  2.448 |
| ACT + GEN | 0.967  0.865  0.929  1.103  0.924  1.012 | 0.151  0.145  0.159  0.133  0.15  0.137 | 1.963  2.014  2.206  2.027  1.937  2.050 |
| ACT + MTX | 0.900  0.969  1.025  0.982  0.985  1.011 | 0.150  0.148  0.143  0.146  0.141  0.152 | 2.581  2.565  2.520  2.585  2.311  2.478 |
| TNF-α | 1.374  1.138  1.508  1.302  1.272  1.330 | 0.145  0.145  0.136  0.141  0.143  0.146 | 2.517  2.466  2.608  2.589  2.455  2.418 |
| TNF-α + GEN | 1.189  1.157  1.254  1.202  1.252  1.051 | 0.134  0.126  0.142  0.132  0.155  0.124 | 2.374  2.353  2.342  2.267  2.317  2.361 |
| TNF-α + MTX | 0.834  1.086  0.867  1.096  0.770  0.837 | 0.144  0.158  0.149  0.136  0.134  0.139 | 2.387  2.442  2.577  2.397  2.340  2.241 |
| LPS | 0.322  0.289  0.335  0.365  0.301  0.412 | 0.133  0.127  0.144  0.139  0.141  0.146 | 0.466  0.469  0.507  0.434  0.479  0.424 |
| LPS + GEN | 0.202  0.179  0.239  0.186  0.184  0.200 | 0.151  0.17  0.155  0.158  0.16  0.156 | 0.293  0.274  0.235  0.220  0.244  0.207 |
| LPS + MTX | 0.208  0.199  0.195  0.236  0.197  0.216 | 0.147  0.126  0.164  0.149  0.16  0.143 | 0.303  0.293  0.309  0.258  0.232  0.320 |
